# Diagnostic Test Accuracy of Deep Learning Prediction Models on COVID-19 Severity: Systematic Review and Meta-Analysis

**DOI:** 10.2196/46340

**Published:** 2023-07-21

**Authors:** Changyu Wang, Siru Liu, Yu Tang, Hao Yang, Jialin Liu

**Affiliations:** 1 Department of Medical Informatics West China Medical School Sichuan University Chengdu China; 2 West China College of Stomatology Sichuan University Chengdu China; 3 Department of Biomedical Informatics Vanderbilt University Medical Center Nashville, TN United States; 4 Xiangya School of Medicine Central South University Changsha China; 5 Information Center West China Hospital Sichuan University Chengdu China

**Keywords:** COVID-19, deep learning, prognostics and health management, Severity of Illness Index, accuracy, AI, prediction model, systematic review, meta-analysis, disease severity, prognosis, digital health intervention

## Abstract

**Background:**

Deep learning (DL) prediction models hold great promise in the triage of COVID-19.

**Objective:**

We aimed to evaluate the diagnostic test accuracy of DL prediction models for assessing and predicting the severity of COVID-19.

**Methods:**

We searched PubMed, Scopus, LitCovid, Embase, Ovid, and the Cochrane Library for studies published from December 1, 2019, to April 30, 2022. Studies that used DL prediction models to assess or predict COVID-19 severity were included, while those without diagnostic test accuracy analysis or severity dichotomies were excluded. QUADAS-2 (Quality Assessment of Diagnostic Accuracy Studies 2), PROBAST (Prediction Model Risk of Bias Assessment Tool), and funnel plots were used to estimate the bias and applicability.

**Results:**

A total of 12 retrospective studies involving 2006 patients reported the cross-sectionally assessed value of DL on COVID-19 severity. The pooled sensitivity and area under the curve were 0.92 (95% CI 0.89-0.94; *I*^2^=0.00%) and 0.95 (95% CI 0.92-0.96), respectively. A total of 13 retrospective studies involving 3951 patients reported the longitudinal predictive value of DL for disease severity. The pooled sensitivity and area under the curve were 0.76 (95% CI 0.74-0.79; *I*^2^=0.00%) and 0.80 (95% CI 0.76-0.83), respectively.

**Conclusions:**

DL prediction models can help clinicians identify potentially severe cases for early triage. However, high-quality research is lacking.

**Trial Registration:**

PROSPERO CRD42022329252; https://www.crd.york.ac.uk/prospero/display_record.php?ID=CRD 42022329252

## Introduction

COVID-19 is a novel, highly contagious disease caused by SARS-CoV-2 [1]. COVID-19 has caused an unprecedented global pandemic in terms of size, transmission, severity, and mortality [2]. As of October 28, 2022, more than 62.6 million cases had been confirmed, including over 6.56 million deaths (World Health Organization [WHO] report) [3]. The dramatic increase in patients with COVID-19 has overwhelmed health care systems worldwide. A critical step in the management of patients with COVID-19 is the accurate assessment and prediction of disease severity, which helps health care providers prioritize resources and improve outcomes [4]. However, early and accurate assessment and prediction of patient severity is a major challenge for physicians.

To help physicians improve the efficiency and accuracy of assessing and predicting the severity of patients, artificial intelligence technology has important applications in this field [5]. With the rapid development of deep learning (DL), more powerful graphics processors have been used in medical image analysis [6]. Some excellent DL frameworks, such as ResNet [7], U-Net [8], DenseNet [9], ScanNet [10], and CapsNet [11], have proven to be useful tools in COVID-19 diagnosis and prediction [12]. Previous systematic reviews have demonstrated that DL-based imaging analysis is more effective than manual analysis in detecting and differentiating COVID-19 [13,14] and in predicting the risk of patient mortality [15,16]. Although these studies illustrate the accuracy of DL in diagnosing COVID-19 and predicting mortality [17], no systematic review has confirmed that DL is effective in assessing and predicting the severity of COVID-19.

The “prediction models” contain both diagnostic prediction models and prognostic prediction models. Diagnostic prediction models are used to assess COVID-19 severity cross-sectionally, whereas prognostic prediction models are used to predict disease severity longitudinally [18]. We conducted this systematic review and meta-analysis to summarize the value of DL prediction models in assessing and predicting COVID-19 severity. These findings will contribute to the application of DL in assessing and predicting the severity of COVID-19 patients.

## Methods

### Study Design

The review was performed according to the PRISMA (Preferred Reporting Items for Systematic Reviews and Meta-Analyses) guidelines and flowchart [19,20] and the PRISMA diagnostic test accuracy checklist (Multimedia Appendix 1) [21]. It was registered in the PROSPERO database (registration number: CRD42022329252).

### Search Strategy and Selection Criteria

We searched PubMed, Scopus, LitCovid, Embase (using the OVID platform), and the Cochrane Library (CENTRAL) from December 1, 2019, to April 30, 2022. The search included terms related to COVID-19, DL, and disease severity (Textbox S1 in Multimedia Appendix 2). In addition, another person independently collected literature through citation searches. After removing duplicates, 2 reviewers (CW and YT) independently performed an initial screening of titles and abstracts using Endnote X9 (Clarivate) software and then independently assessed articles against the inclusion criteria using Zotero software (Corporation for Digital Scholarship). Disagreements were resolved by discussion and, where necessary, by third-party adjudication.

The inclusion criteria were (1) evaluating the assessment or predictive value of DL algorithms on disease severity in patients with COVID-19; (2) disclosing the code of the DL algorithm or detailing the parameters used by the model, such as training epochs, learning rate, batch, optimizer, validation strategy, and so forth; (3) reconstructing a 2×2 confusion matrix from sensitivity, specificity, positive predictive value, and negative predictive value; and (4) peer-reviewed articles. Reviews, protocols, and editorials were excluded. Studies that did not clearly indicate the source of the patient data sets were also excluded.

### Quality Assessment

The QUADAS-2 (Quality Assessment of Diagnostic Accuracy Studies 2) criteria assessed the risk of bias in 4 domains: patient selection, index test, reference standard, and flow and timing. However, QUADAS-2 cannot be evaluated against predictive models for diagnosis or prognosis [22], and to refine this, we further introduced the PROBAST (Prediction Model Risk of Bias Assessment Tool) [23], which is well suited to address DL predictive models for binary outcomes [24]. Furthermore, PROBAST assessed the risk of bias in 4 other domains: participants, predictors, outcomes, and analysis.

### Data Analysis

Statistical analysis was performed with STATA (version 17.0) using the MIDAS module [25] and the METAPROP module [26]. Postestimation procedures for model diagnostics and empirical Bayesian predictions were used to assess heterogeneity using the *I*^2^ statistic. The following metrics were used: 0%-40% (not important heterogeneity), 30%-60% (moderate heterogeneity), 50%-90% (substantial heterogeneity), and 75%-100% (considerable heterogeneity) [27]. Deek funnel plots were tested for publication bias using an asymmetry test. If *P*<.10, publication bias may be present. Using bivariate mixed-effects logistic regression modeling [25], forest plots were used to compare the sensitivity and the specificity of DL models for assessing and predicting disease severity in patients with COVID-19. Summary receiver operating characteristic (SROC) curves were adopted to assess overall diagnostic accuracy. The Fagan nomogram was used to explore the relationship between pretest probability, likelihood ratio (LR), and posttest probability. LR dot plots, divided into 4 quadrants based on the strength of the evidence threshold, were used to determine the exclusion and confirmation of the DL model. Finally, subgroup analyses were performed to examine whether the estimated sensitivity, specificity, and associated *I*^2^ (when each subgroup included 4 or more studies) differed by a number of moderators. Details are provided in Textbox S2 in Multimedia Appendix 2.

## Results

### Search Outcome

**Figure 1:**
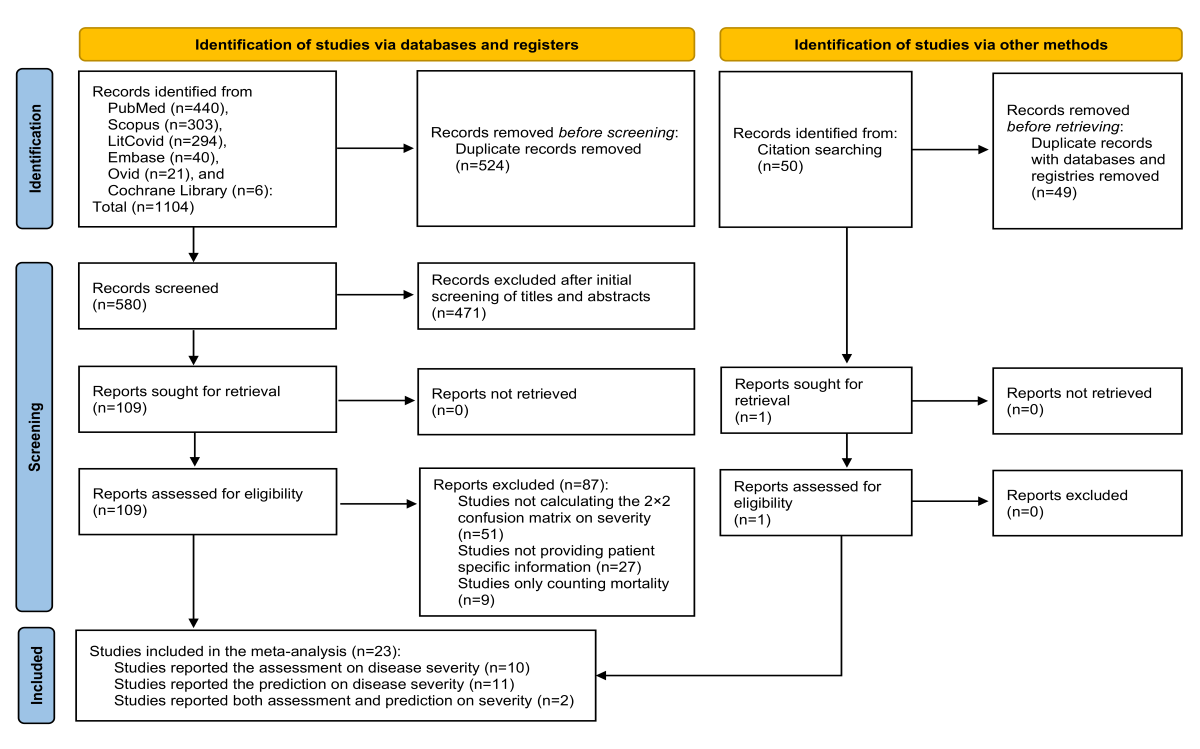
PRISMA (Preferred Reporting Items for Systematic Reviews and Meta-Analyses) flowchart of the review process and study selection.

A total of 1154 titles and abstracts were identified in the initial search. According to this study’s inclusion and exclusion criteria, 1044 articles were excluded. In addition, 110 studies were reviewed for full text, of which 23 met all inclusion criteria (Figure 1).

The PRISMA 2020 flowchart for new systematic reviews included searches of databases, registers, and other sources [20].

### Study Characteristics

All studies were retrospective and used completely different data sources. Eleven of these studies classified the stage as severe or critical according to the guidelines for diagnosis and treatment of COVID-19 infection from the National Health Commission of the People’s Republic of China [28-38]. However, except for the study in which disease was determined by scoring the image parameters [39,40], all other studies defined severe patients as having at least one of the following criteria: respiratory rate ≥30 breaths/min, oxygen saturation ≤93% at rest, PaO_2_/FiO_2_ ≤300 mmHg, significant progression of pulmonary lesions (>50%) within 24-48 h, mechanical ventilation, intensive medical care, or shock. Details of the criteria for severe patients in different studies, study type, and the design characteristics of the DL model are provided in Tables S1 and S2 in Multimedia Appendix 2. Table 1 summarizes the characteristics of the included studies and the diagnostic test accuracy of the DL prediction model.

**Table 1:** Characteristics of the studies included in the meta-analysis.

Study			Deep learning model		Input: imaging data^a^		Input: no medical imaging		Model performance: optimizer / validation strategies / interpretability		Partition/No. of patients (severe) / area under curve		2×2 Truth table: true positive / false negative / true negative / false positive
**Assessment**													
	Cai et al 2020 [28]	UNet		Chest CT^b^		Age, LYC^c^, NEC^d^, PaO_2_^e^, SaO_2_^f^		Mini-batch + Adam / cross-validation (10-fold, 100 repetitions) / N/A^g^		ET^h^ / 99 (74) / 0.93		67 / 7 / 20 / 5	
	Carvalho et al 2020 [39]	ANN^i^		Chest CT		None		N/A / cohort validation / quantitative results		IT^j^ / 97(35) / 0.90		31 / 4 / 61 / 1	
	Li et al 2020 [29]	2D UNet + ResNet-34		Chest CT		None		N/A / N/A / N/A		ET / 196 (32) / 0.97		30 / 2 / 144 / 20	
	Xiao et al 2020 [37]	ResNet-34		Initial chest CT		None		N/A / cross-validation (5-fold) / N/A		ET / 105 (40) / 0.89		35 / 5 / 51 / 14	
	Yu et al 2020 [30]	DenseNet-201		Chest CT		None		N/A / cross-validation (10-fold) / N/A		IT / 40 (13) / 0.99		12 / 1 / 26 / 1	
	Aboutalebi et al 2021 [40]	COVIDNet		CXR^k^		None		Adam / radiologist validation / GSInquire		IT / 150 (98) / 0.96		91 / 7 / 48 / 4	
	Feng et al 2021 [38]	UNet++		Chest CT		Cardiovascular or cerebrovascular diseases, COPD^l^, diabetes, hs-Cardiac troponin I, hypertension, LDH^m^		Grid search / cross-validation (5-fold) / N/A		ET / 98(8) / 0.97		8 / 1 / 77 / 13	
	He et al 2021 [31]	UNet		3D chest CT		None		SGD^n^ / cross-validation (5-fold) / N/A		IT / 191(51) / 0.99		49 / 2 / 190 / 1	
	Ho et al 2021 [41]	ResNet-50 + InceptionV3 + DenseNet121 + ANN		3D CT		CRP^o^, SaO_2_, respiratory rate, systolic blood pressure, WBC^p^ count		Adam + binary cross-entropy / cross-validation (5-fold) / gradient-weighted class activation mapping		IT / 58 (7) / 0.92		6 / 1 / 49 / 2	
	Li et al 2021 [32]	CNN^q^		Chest CT		None		Adam / cross-validation (10-fold) / predicted label + visualization of the attention mechanism		IT / 229 (50) / 0.98		47 / 3 / 173 / 6	
	Udriștoiu et al 2021 [42]	VGG-19 + ResNet-50 + DenseNet-121 + InceptionV3		CXR		None		Adam + root mean square propagation / cross-validation (5-fold) / selector control box testing data set		IT / 95 (35) / 0.98		34 / 1 / 60 / 0	
	Ortiz et al 2022 [43]	DenseNet-161		Chest CT		None		Root mean square propagation / cross-validation (5-fold)/ N/A		IT / 596 (107) / N/A		95 / 12 / 470 / 19	
**Prediction**													
	Ning et al 2020 [33]	InceptionV3 + DenseNet-121 + VGG-16		Chest CT		Age, albumin, alanine aminotransferase, aspartate aminotransferase, brain natriuretic peptide, CD4+ T cell, calcium, creatinine, CRP, eosinophil count, globulin, γ-Glutamyl transpeptidase, LYC, monocyte count, NEC, platelet, procalcitonin, sex, sodium, total bilirubin, urea, WBC count		Adam / cross-validation (10-fold, 10 repetitions) / N/A		ET / 252 (63) / 0.88		50 / 13 / 148 / 41	
	Xiao et al 2020 [37]	ResNet-34		Initial chest CT		None		N/A / cross-validation (5-fold) / N/A		ET / 65 (11) / 0.92		9 / 2 / 42 / 12	
	Zhang et al 2020 [44]	UNet + FCN^r^ + DeepLabv3 + ResNet-18		Chest CT		Age, albumin, activated partial thromboplastin time, CRP, indirect bilirubin, LDH, LYC, NEC, platelet count, respiratory rate, SaO_2_, temperature, thrombin time, Na^+^, K^+^, HCO_3_^–^		SGD + Adam / cross-validation (5-fold) / SHAP^s^		IT / 432 (158) / 0.91		126 / 32 / 238 / 36	
	Fang et al 2021 [34]	3D ResNet		Chest CT		Albumin, aspartate aminotransferase, brain natriuretic peptide, CD3^+^CD4^+^T cells count, CRP, creatinine, fever, γ-Glutamyl transpeptidase, hypertension, troponin, WBC count		Adam / cross-validation (5-fold) / gradient-weighted class activation mapping		IT / 363 (154) / 0.89 ET / 133 (54) / 0.86		IT: 117 / 37 / 175 / 34; ET: 40 / 14 / 68 / 11	
	Feng et al 2021 [38]	UNet++		Chest CT		Cardiovascular or cerebrovascular diseases, COPD, diabetes, hs-Cardiac troponin I, hypertension, LDH		Grid search / cross-validation (5-fold) / N/A		ET / 98 (8) / 0.88		6 / 2 / 79 / 11	
	Jiao et al 2021 [45]	UNet + VGG-11 + EfficientNet-B0		CXR		Age, cardiovascular disease, chronic kidney disease, chronic liver disease, COPD, creatinine, CRP, diabetes, fever, hypertension, LYC, malignant tumor, sex, SpO_2_^t^, WBC count		N/A / cohort validation / N/A		IT / 366 (84) / 0.85; ET / 475 (125) / 0.79		IT: 62 / 22 / 241 / 41; ET: 91 / 34 / 245 / 105	
	Kwon et al 2021 [46]	DenseNet-121		CXR		None		Adam + binary cross-entropy / cohort validation / N/A		IT / 156 (46) / 0.88		38 / 8 / 78 / 32	
	Lassau et al 2021 [47]	ResNet50 + EfficientNetB0 + UNet		Chest CT		Age, platelet count, SaO_2_, sex, urea		N/A / cross-validation (5-fold) / logistic regression		IT / 150 (44) / 0.76		31 / 13 / 80 / 26	
	Shi et al 2021 [35]	VNet		Chest CT		Age, CD4^+^ T cell count, CRP, LDH		N/A / cross-validation (10-fold) / N/A		IT / 196 (45) / 0.90		36 / 9 / 130 / 21	
	Soda et al 2021 [48]	UNet + ResNet-50		CXR		Age, D-dimer, diabetes, LDH, sex, SaO_2_, WBC count		Adam + SGD / cross-validation (10-fold, 20 repetitions) / N/A		IT / 820 (436) / N/A		325 / 111 / 288 / 96	
	Chieregato et al 2022 [49]	3D CNN		Chest CT		Age, creatinine, creatine kinase		Optuna + SGD / Cross-validation (10-fold) / SHAP analysis		IT / 107 (31) / 0.95		26 / 5 / 71 / 5	
	Chen et al 2022 [36]	Mask R-CNN + ANN		Chest CT		Hematocrit, LYC, NEC, platelet count, red blood cell count		N/A / cross-validation (10-fold) / statistical analysis of clinical data		IT / 140 (70) / 0.76		55 / 15 / 51 / 19	
	Wang et al 2022 [50]	EfficientNet		Chest CT		Age, cancer, cardiovascular disease, chronic kidney disease, chronic liver disease, COPD, diabetes, fever, hypertension, HIV, LYC, sex, WBC count		N/A / cohort validation / N/A		IT / 209 (45) / 0.86		33 / 12 / 146 / 18	

^a^Imaging data include total lesion volume, volume change, proportion of lesions, mean density, edge clarity, pleural distance, form, mean lesion volume, MOICT, lesion range score, number of segments involved, CT/CXR severity score, consolidation, and ground-glass opacification.

^b^CT: computed tomography.

^c^LYC: lymphocyte count.

^d^NEC: neutrophil count.

^e^PaO_2:_ partial pressure of oxygen.

^f^SaO_2:_ oxygen saturation.

^g^N/A: not available.

^h^ET: external test.

^i^ANN: artificial neural network.

^j^IT: internal test.

^k^CXR: chest x-ray.

^l^COPD: chronic obstructive pulmonary disease.

^m^LDH: lactate dehydrogenase.

^n^SGD: stochastic gradient descent.

^o^CRP: C-reactive protein.

^p^WBC: white blood cell.

^q^CNN: convolutional neural network.

^r^FCN: fully connected neural network.

^s^SHAP: Shapley Additive Explanations.

^t^SpO2: oxygen saturation

### Outcomes of DL Models for COVID-19 Severity

#### Cross-Sectional Assessment

**Figure 2:**
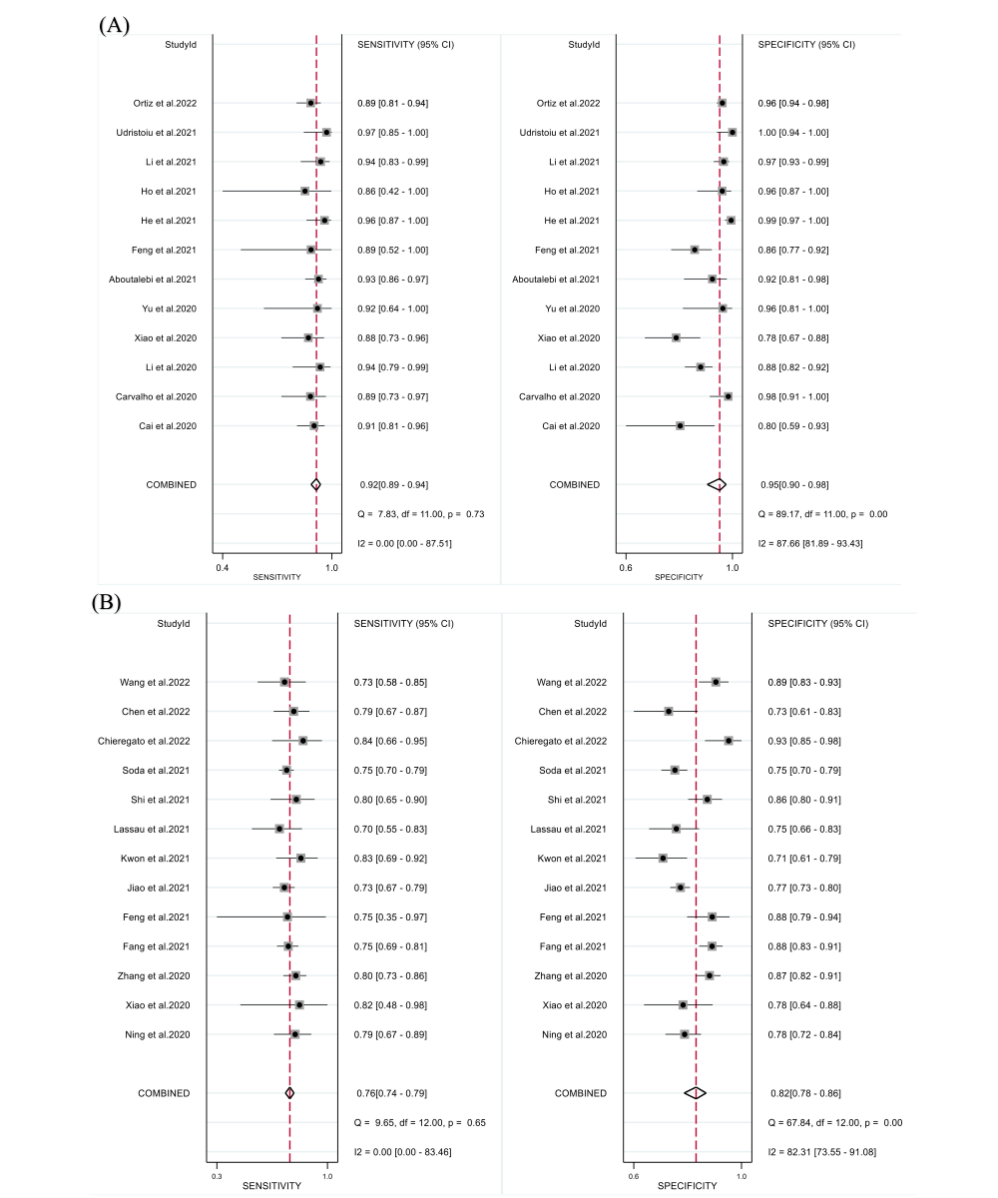
Forest plots in sensitivity and specificity of DL models. (A) Assessing disease severity in patients with COVID-19. The pooled sensitivity and specificity were 0.92 (95% CI 0.89-0.94) and 0.95 (95% CI 0.90-0.98), respectively [28-32,37-43]. (B) Predicting disease severity in patients with COVID-19. The pooled sensitivity and specificity were 0.76 (95% CI 0.74-0.79) and 0.82 (95% CI 0.78-0.86), respectively [33-38,44-50].

**Figure 3:**
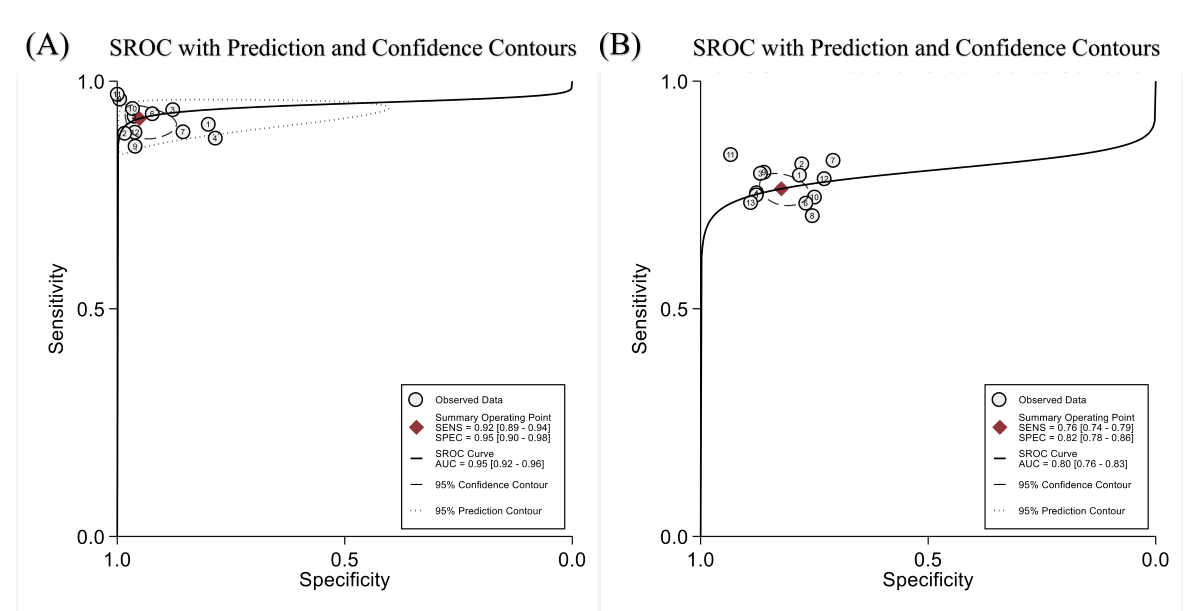
The SROC graph for the studies. (A) The AUC of deep learning (DL) models for assessing disease severity was 0.95 (95% CI 0.92-0.96). (B) The AUC of DL models for predicting disease severity was 0.80 (95% CI 0.76-0.83). AUC: area under the curve; SENS: sensitivity; SPEC: specificity; SROC: summary receiver operating characteristic.

A total of 12 studies with 2006 patients reported the assessment value of DL models for disease severity. The pooled sensitivity and specificity were 0.92 (95% CI 0.89-0.94; *I*^2^=0.00%) and 0.95 (95% CI 0.90-0.98; *I*^2^=87.66%), respectively (Figure 2). The diagnostic odds ratio, the positive likelihood ratio (LR^+^), and the negative likelihood ratio (LR^–^) were 217 (95% CI 89-532), 18.8 (95% CI 9.3-38.0), and 0.09 (95% CI 0.06-0.12), respectively. In the SROC curve (Figure 3), the area under the curve of DL models for assessing disease severity was 0.95 (95% CI 0.92-0.96), indicating a high diagnostic value.

**Figure 4:**
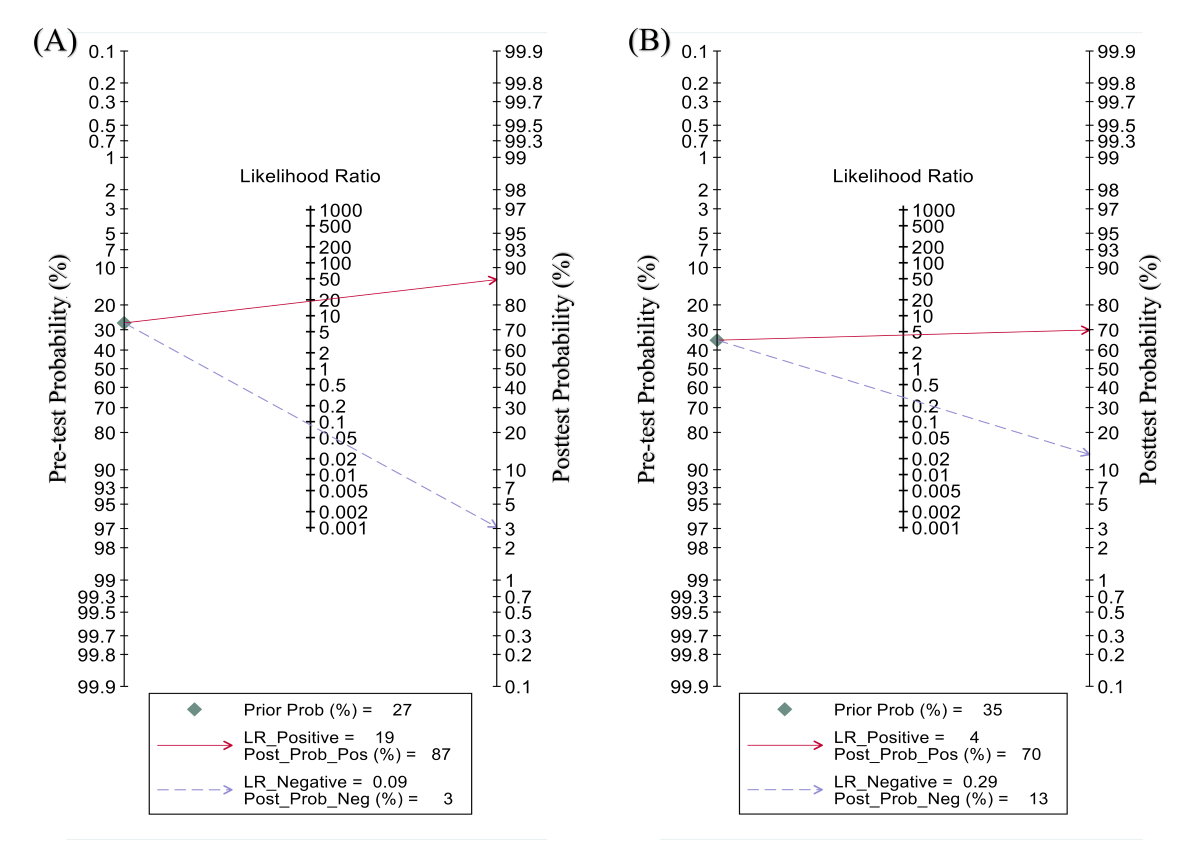
Fagan nomogram of deep learning (DL) models for assessing and predicting disease severity in patients with COVID-19. The first column of this nomogram represents the pre-test probability, the second column represents the Likelihood Ratio, and the third shows the posttest probability. The pre-test probabilities were set to 27% and 35%, respectively. (A) The post-test probability of DL models for the assessment of severe cases was 87% when the Pretest Prob of Disease was above the cut-off value. The post-test probability was 3% when the Pretest Prob of Disease was below the cut-off value. (B) The post-test probability of DL models for the prediction of severe cases was 70% when the Pretest Prob of Disease was above the cut-off value. The post-test probability was 13 % when the Pretest Prob of Disease was below the cut-off value.

**Figure 5:**
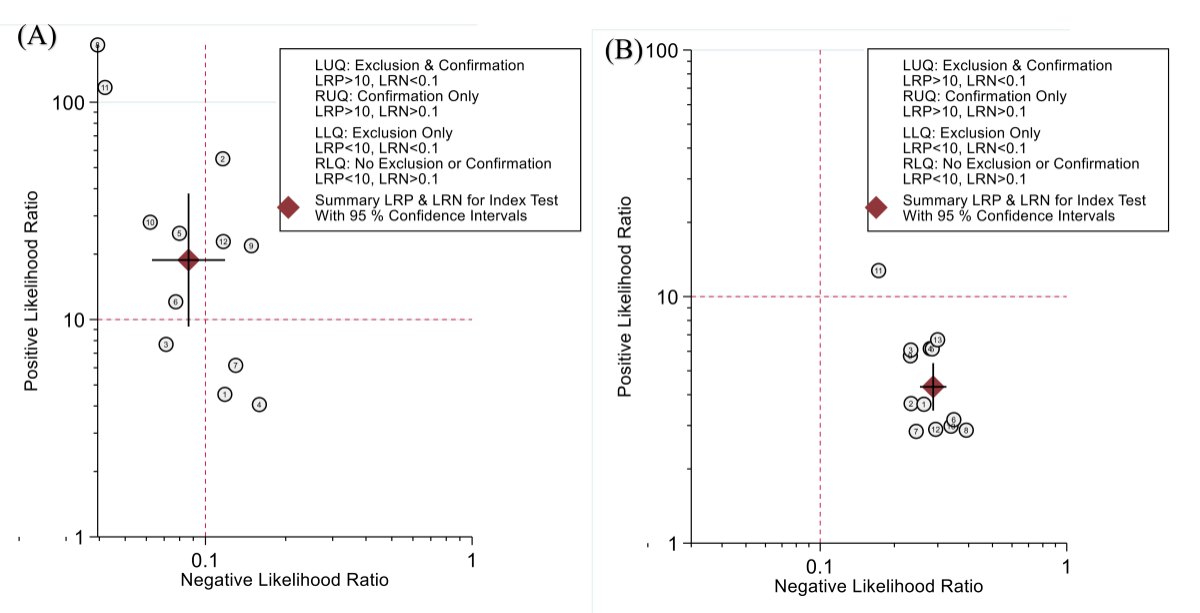
Likelihood ratio dot plot of deep learning (DL) prediction models. (A) The summary point of DL models for assessing severe cases was in the left upper quadrant (LR+ >10 and LR– <0.1: exclusion and confirmation) [51]. (B) The summary point of DL models for predicting severe cases was in the right lower quadrant (LR+ <10 and LR– >0.1: no exclusion or confirmation). LRN: negative likelihood ratio; LRP: positive likelihood ratio; LUQ: left upper quadrant; RLQ: right lower quadrant; RUQ: right upper quadrant.

Based on the Pretest Probability of Disease [25], we set the pretest probability to 27%. At this point, true positive accounted for 87% when patients were diagnosed with severe COVID-19 by the DL model, and false negative accounted for 3% when the diagnosis was nonsevere disease (Figure 4). DL models for assessing disease severity produced a conclusive change in probability from pretest to posttest (Figure 5) [51].

The first column of this nomogram represents the pretest probability, the second column represents the likelihood ratio, and the third column shows the posttest probability. The pretest probabilities were set to 27% and 35%, respectively. The posttest probability of DL models for the assessment of severe cases was 87% when the Pretest Probability of Disease was above the cut-off value. The posttest probability was 3% when the Pretest Probability of Disease was below the cutoff value. The posttest probability of DL models for the prediction of severe cases was 70% when the Pretest Probability of Disease was above the cutoff value. The posttest probability was 13% when the Pretest Probability of Disease was below the cutoff value.

#### Longitudinal Prediction

A total of 13 studies with 3951 patients reported the predictive value of DL models for disease severity. The pooled sensitivity and specificity were 0.76 (95% CI 0.74-0.79; *I*^2^=0.00%) and 0.82 (95% CI 0.78-0.86; *I*^2^=82.32%), respectively (Figure 2). The diagnostic odds ratio, the LR^+^, and the LR^–^ were 15 (95% CI 11-21), 4.3 (95% CI 3.4-5.4), and 0.29 (95% CI 0.25-0.33), respectively. In the SROC curve (Figure 3), the area under the curve of the DL models for predicting disease severity was 0.80 (95% CI 0.76-0.83).

Based on the Pretest Probability of Disease [25], we set the pretest probability at 35%. At this point, if admitted patients were judged by the DL model to be progressing to severe COVID-19, the probability of TP was 70%, and if they were judged not to progress to severe disease, the probability of FN was 13% (Figure 4). The likelihood ratio plot (Figure 5) shows that the DL models used to predict disease severity produced small changes [51].

### Methodological Quality

#### QUADAS-2

**Figure 6:**
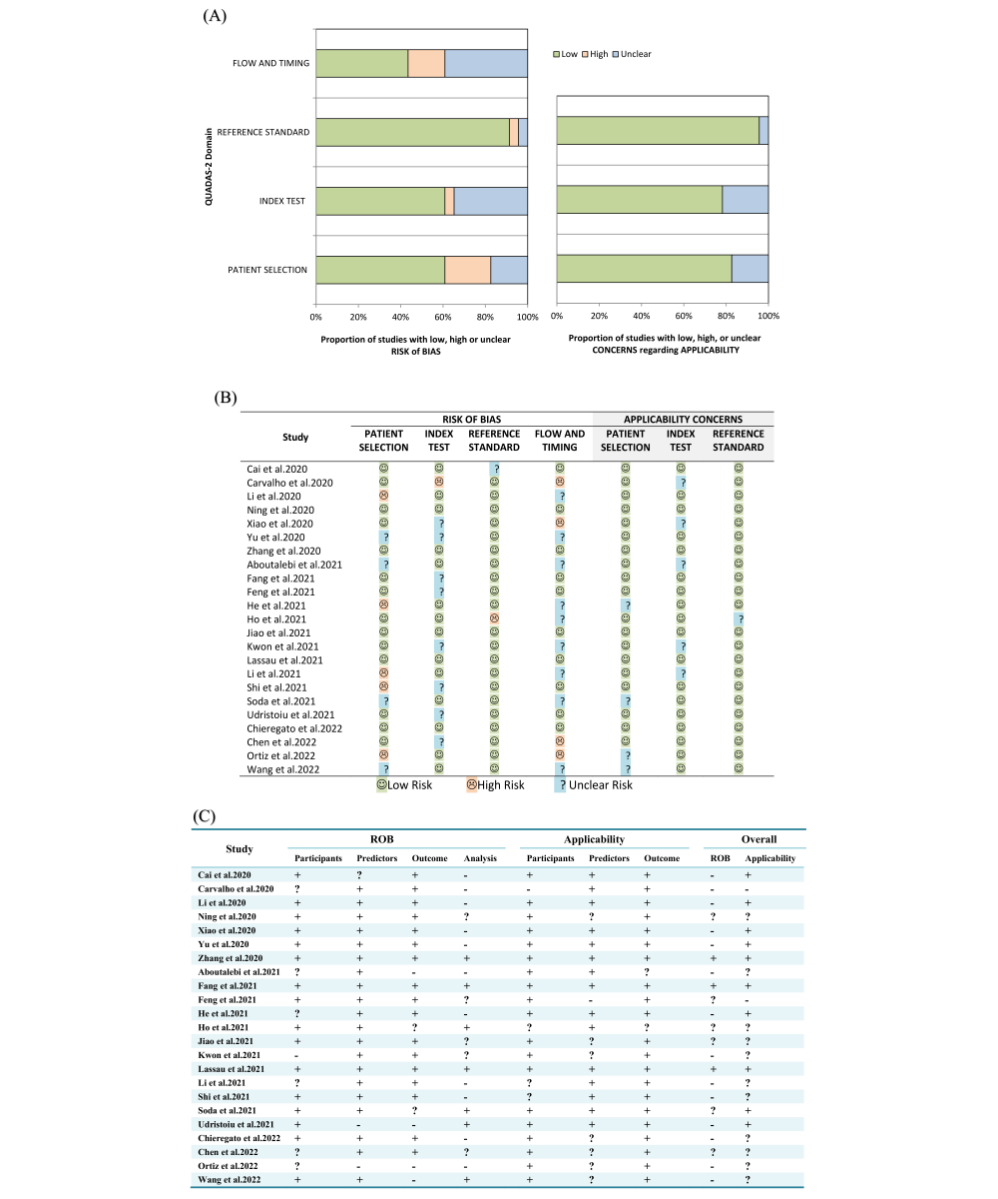
Methodological assessment by QUADAS-2 and PROBAST. (A) Proportion of risk of bias for all domains and proportion of applicability concerns in 3 domains. (B) Summary of the risk of bias for each study. Green, blue, and red circles represent a low, unclear, and high risk of bias, respectively. (C) Tabular presentation for PROBAST results. The “+” indicates low ROB (risk of bias) or low concern regarding applicability, “-” indicates high ROB or high concern regarding the applicability, and “?” indicates unclear ROB or unclear concern regarding the applicability.

Regarding the QUADAS-2 risk of bias assessment (Figure 6), we found 9 studies with a high risk of bias [29,31,32,35-37,39,41,43], 16 studies with an unclear risk of bias [28-32,34-38,40-42,46,48,50], and 5 studies with a completely low risk of bias [33,44,45,47,49]. In particular, 5 of the included studies did not report details of patient selection [29,31,32,35,43], and 4 provided unclear information on patient selection [30,40,48,50], resulting in a high and unclear bias in patient selection. Moreover, the threshold was not prespecified in one study [39], leading to a high risk of bias in the index test, and 8 studies provided unclear information on how to perform the index test [30,34-38,42,46], leading to an unclear risk of bias. Furthermore, one study interpreted the results of reference standards when the results of the index test were known [41], leading to a high risk of bias in the reference standard, and another did not explain this [28], which was considered to be an unclear risk of bias. In addition, 4 studies used reference standards for indicator tests [36,39,43], or did not include all patients in the study [37], resulting in high process and time bias. The other 9 articles did not provide clear information, resulting in unclear [29-32,40,41,46,48,50].

#### PROBAST

After evaluating the predictive models using PROBAST (Figure 6), we found 14 [28-32,35,37,39,40,42,43,46,49,50], 6 [33,36,38,41,45,48], and 3 studies [34,44,47] with high, unclear, and low risk of bias, respectively. Moreover, 2 [38,39], 11 [32,33,35,36,40,41,43,45,46,49,50], and 10 studies [28-31,34,37,42,44,47,48] were of high, unclear, and low concern for applicability, respectively. However, only 3 studies [34,44,47] had both a low risk of bias and a low concern about applicability. In terms of the risk of bias, the selection of predictors based on univariate analysis was the main source of risk, causing 11 high risks [28-32,35,37,39,40,43,49] and 5 unclear risks [33,36,38,45,46]. In contrast, for applicability, the main concern was with the predictor variables, causing 1 high concern [38] and 7 unclear concerns [33,36,43,45,46,49,50].

We found the overall quality of the included studies to be poor, with only 2 studies having a low risk of bias in both QUADAS-2 and PROBAST [44,47].

#### Publication Bias

Two funnel plots were also used to assess the publication bias for each of the 23 studies that met the inclusion criteria. Deek funnel plots are shown in Figure S1 in Multimedia Appendix 2. According to Sterne [52], when publication bias is very low, the points are distributed symmetrically around the true effect. Publication bias was low in studies reporting the assessed value of DL models for disease severity (*P*=.61) and the predictive value of DL models for disease severity (*P*=.22).

### Subgroup Analyses

We performed the subgroup analyses in 6 areas, including data partition (internal test or external test), data sources (single benchmark or multiple benchmark), training method (pretrained or customized), DL model networks (ResNet or other networks), input parameters (image parameters only or clinical and image parameters), and image (computed tomography [CT] or x-ray), to effectively understand how the different 6 types affected the performance of the algorithm for COVID-19 assessment and prediction.

In sensitivity, from univariable meta-regression and subgroup analyses (Figure S2 in Multimedia Appendix 2), we can learn that all domains influenced the heterogeneities of sensitivity for assessing and predicting disease severity, but none of the 6 influenced the DL model for assessing and predicting COVID-19 severity (Table 2), as their heterogeneities were very low (*I*^2^=0.00%).

In terms of data partitioning, the specificity of the internal test and external test data sets for assessing disease severity was 0.98 and 0.85, respectively, with significant heterogeneity between groups (*P*<.001). On the other hand, subgroups based on sources (*P*=.001), training method (*P*=.01), input parameter (*P*=.02), or image (*P*<.001) may have intergroup heterogeneity in the specificity of prediction. Among them, the specificity of 0.90 for a single source was higher than that of 0.80 for a multicenter. Furthermore, the customized training method achieves a specificity of 0.87, while the pretraining method achieves only 0.80. Additionally, the specificity of the parameter that included both clinical and image data was 0.83, while the parameter that included only image data was 0.73. Finally, the specificity of the DL model using x-ray was 0.78, which was significantly lower than the specificity of the model using CT, which was 0.84. Detailed results of the subgroup analyses are shown in Table 2, and corresponding plots are presented in Figure S3 in Multimedia Appendix 2.

**Table 2:** Results of sensitivity analysis.

Categories			Studies, n		Sensitivity (95% CI)	*I*^2^ (%)	*P* value (HBG^a^ of sensitivity)		Specificity (95% CI)		*I*^2^ (%)		*P* value (HBG of specificity)	
**Assessment**														
	**Data partition**							.30						<.001
		Internal test		8	0.94 (0.91-0.96)	0.00			0.98 (0.96-0.99)		50.96			
		External test		4	0.91 (0.86-0.95)	0.00			0.85 (0.80-0.89)		18.38			
	**Data sources**							.31						.93
		Single		4	0.94 (0.91-0.98)	0.00			0.94 (0.85-0.99)		87.54			
		Multiple		8	0.92 (0.89-0.95)	0.00			0.94 (0.89-0.98)		84.93			
	**Training method**							.11						.33
		Pretrained		5	0.90 (0.85-0.94)	0.00			0.92 (0.81-0.98)		79.68			
		Customized		7	0.94 (0.91-0.96)	0.00			0.95 (0.91-0.98)		86.90			
	**DL^b^ model networks**							.46						.60
		ResNet		4	0.94 (0.90-0.99)	4.58			0.93 (0.81-0.99)		88.56			
		Other networks		8	0.92 (0.90-0.95)	0.00			0.95 (0.91-0.98)		79.38			
	**Input parameter**							.34						.12
		Only image parameter		9	0.93 (0.91-0.96)	0.00			0.95 (0.91-0.98)		85.32			
		Clinical and image parameter		3	0.90 (0.84-0.96)	N/A^c^			0.89 (0.78-0.96)		N/A			
	**Image**							.23						.10
		CT^d^		10	0.92 (0.89-0.95)	0.00			0.93 (0.89-0.97)		94.32			
		X-ray		2	0.95 (0.91-0.99)	N/A			0.98 (0.94-1.00)		N/A			
**Prediction**														
	**Data partition**							.68						.52
		Internal test		10	0.77 (0.74-0.79)	0.00			0.83 (0.78-0.87)		82.85			
		External test		5	0.75 (0.70-0.81)	0.00			0.80 (0.73-0.87)		82.60			
	**Data sources**							.21						.001
		Single		2	0.82 (0.73-0.90)	N/A			0.90 (0.86-0.94)		N/A			
		Multiple		11	0.76 (0.74-0.78)	0.00			0.80 (0.76-0.84)		79.75			
	**Training method**							.19						.01
		Pretrained		10	0.76 (0.73-0.78)	0.00			0.80 (0.75-0.84)		83.96			
		Customized		3	0.80 (0.74-0.85)	N/A			0.87 (0.84-0.90)		N/A			
	**DL model networks**							.53						.62
		ResNet		5	0.76 (0.73-0.79)	0.00			0.80 (0.75-0.86)		79.85			
		Other networks		8	0.77 (0.74-0.81)	0.00			0.82 (0.77-0.88)		85.84			
	**Input parameter**							.20						.02
		Only image parameter		2	0.82 (0.73-0.92)	N/A			0.73 (0.67-0.80)		N/A			
		Clinical and image parameter		11	0.76 (0.73-0.78)	0.00			0.83 (0.79-0.87)		84.96			
	**Image**							.24						<.001
		CT		10	0.78 (0.75-0.81)	0.00			0.84 (0.81-0.88)		70.06			
		X-ray		3	0.75 (0.71-0.79)	N/A			0.76 (0.73-0.78)		N/A			

^a^HBG: heterogeneity between group.

^b^DL: deep learning.

^c^N/A: not available.

^d^CT: computed tomography.

## Discussion

### Model Performance

Among the DL models included in the systematic review, CT was used more frequently than x-ray: CT was used in 10 of the DL models assessing COVID-19 severity and in 10 of the models predicting severity. However, there is no significant difference in their impact on model performance.

After evaluating sensitivity, specificity, and LR together [53], we found that DL achieved higher sensitivity and specificity in assessing the severity of COVID-19 compared to using CT [54] or neutrophil-lymphocyte ratio (NLR) alone [55]. However, DL models for longitudinal prediction of disease severity failed to exclude and confirm patients. Although the DL model was significantly superior to thrombocytopenia in predicting disease progression [56], the results with NLR resembled the ones obtained using DL [57,58].

### Predictor Variables

The parameters used in the DL model should be derived from predictor variables that are known predictors in the scientific literature, thus limiting overfitting [59]. However, only 4 of the 23 articles used this approach to select predictor variables [41,44,45,47]. Of the remaining articles, 10 adopted univariate variables [29-32,37,39,40,42,43,46], and 9 used variables with significant levels in clinical analyses [28,33-36,38,48-50]. However, univariate variables or variables with significant levels in clinical analyses may not be suitable as candidate predictors [60]. We specified a list of candidate predictors (Table S3 in Multimedia Appendix 2), which were summarized in a systematic literature review of prognostic factors affecting COVID-19 prognosis. However, the number of predictors needs to be determined by the sample size [61]. Too many predictor variables may, on the one hand, prevent the model from providing valid estimates in new patients [62] and may include variables that are not relevant to the outcome and lead to test bias [62,63]. This unfavorable situation occurred in 5 of our included studies [33,38,44,49,50].

### Data Sets

Model exploitation requires both a training set (ie, a developmental data set) and a validation set (ie, an internal validation data set) [64]. Once the predictive model is complete, an external test set (ie, an external validation data set) is strongly recommended to evaluate the performance of the model [65], but only 7 articles have done so [28,29,33,34,37,38,45]. The internal test set generated by temporal partitioning (ie, the temporal validation data set) is considered effective as an intermediate between the validation set and the external test set [18]. This approach was used in 3 of the 18 studies that used internal test sets [45-47]. However, the remaining 15 generated internal test sets with random splitting [30-32,34-36,39-44,48-50], which was considered inefficient [64].

### Heterogeneity

The DL prediction model has relatively low heterogeneity with respect to sensitivity but considerable heterogeneity with respect to specificity. As a result of the sensitivity analysis (Table 2), for specificity, the heterogeneity in assessment comes mainly from the data partitioning, whereas the heterogeneity in prediction comes from 5 aspects: data partitioning, data sources, training method, DL model networks, and image. However, there is no significant difference in these 5 aspects, which may be related to the low performance of the vertical prediction model.

The specificity of the external test data set was significantly lower than that of the internal test data set, suggesting that the study warrants external validation [18]. Although there may be intergroup heterogeneity in the specificity of COVID-19 severity prediction based on subgroups of sources, training methods, input parameters, or images, they are all unevenly distributed within their subgroups. Therefore, the impact of these 4 aspects on the specificity of DL prediction models needs to be further investigated. In DL model networks, Komolafe et al [53] found a difference between ResNet and other network models in detecting COVID-19, whereas our study found no significant difference in sensitivity or specificity between ResNet and other network architectures in diagnosing and predicting the severity of COVID-19 (Table 2). This result suggests that, unlike in disease detection, changing the network architecture alone may have little significant impact on DL performance and that factors such as subgroups of sources, training methods, input parameters, and images need to be taken into account.

### Limitations

The study has several limitations. First, all included studies were retrospective, which may introduce bias due to missing information and unavailable confounders [66]. Second, all of these studies lacked large-scale clinical data. Third, although the effect of 6 aspects on the DL model to assess and predict severity was investigated, no further analysis of specific clinical factors, such as NLR and disease process spectrum, was performed [18]. Finally, only 7 articles used external tests [28,29,33,34,37,38,45], and no studies explicitly cited the TRIPOD (Transparent Reporting of a Multivariable Prediction Model of Individual Prognosis or Diagnosis) [64].

### Conclusions

The meta-analysis showed a remarkably high performance of the DL model for assessing COVID-19 severity and good predictive values for disease severity. However, high-quality studies are lacking. We hope that more researchers will take advantage of the upcoming TRIPOD-AI (Transparent Reporting of a Multivariable Prediction Model of Individual Prognosis or Diagnosis–Artificial Intelligence) to standardize their studies on DL or machine learning prediction models [67]. Significantly, the predictive performance of DL for COVID-19 severity leaves much to be desired. This suggests that future studies will require a more rigorous and scientific approach. We suggest using multiple clinical factors that have been confirmed by clinical studies to be associated with COVID-19 severity as predictor variables, dividing the development data set and internal validation data sets according to the time of admission of patients with COVID-19, and using data from other hospitals to assess the performance of the model. However, there is no denying that DL can help clinicians quickly identify patients that are severely ill and detect potentially serious cases early, leading to earlier treatment and more efficient health care systems.

## Appendix

Multimedia Appendix 1 PRISMA DTA (Preferred Reporting Items for Systematic Reviews and Meta-Analyses Extension for Diagnostic Test Accuracy) checklist.

Multimedia Appendix 2 Supplementary information.

## Abbreviations

CT: computed tomography
DL: deep learning
LR: likelihood ratio
NLR: neutrophil-lymphocyte ratio
PRISMA: Preferred Reporting Items for Systematic Reviews and Meta-Analyses
PROBAST: Prediction Model Risk of Bias Assessment Tool
QUADAS-2: Quality Assessment of Diagnostic Accuracy Studies 2
SROC: summary receiver operating characteristic
TRIPOD: Transparent Reporting of a Multivariable Prediction Model of Individual Prognosis or Diagnosis
TRIPOD-AI: Transparent Reporting of a Multivariable Prediction Model of Individual Prognosis or Diagnosis
WHO: World Health Organization
