# Supplementary material for: Diagnostic Test Accuracy of Deep Learning Prediction Models on COVID-19 Severity: Systematic Review and Meta-Analysis
Source: J Med Internet Res. 2023 Jul 21;25:e46340. doi: 10.2196/46340 (PMC10403760; doi:10.2196/46340)
Supplement: Multimedia Appendix 2 [file jmir_v25i1e46340_app2.docx]

**Multimedia Appendix 2**

**Textbox S1. Full search strings.**

| **PubMed**  ("COVID-19"[Mesh] OR (((((((((((((((((((((((((((((((((((COVID 19) OR (SARS-CoV-2 Infection)) OR (Infection, SARS-CoV-2)) OR (SARS CoV 2 Infection)) OR (SARS-CoV-2 Infections)) OR (2019 Novel Coronavirus Disease)) OR (2019 Novel Coronavirus Infection)) OR (2019-nCoV Disease)) OR (2019 nCoV Disease)) OR (2019-nCoV Diseases)) OR (Disease, 2019-nCoV)) OR (COVID-19 Virus Infection)) OR (COVID 19 Virus Infection)) OR (COVID-19 Virus Infections)) OR (Infection, COVID-19 Virus)) OR (Virus Infection, COVID-19)) OR (Coronavirus Disease 2019)) OR (Disease 2019, Coronavirus)) OR (Coronavirus Disease-19)) OR (Coronavirus Disease 19)) OR (Severe Acute Respiratory Syndrome Coronavirus 2 Infection)) OR (SARS Coronavirus 2 Infection)) OR (COVID-19 Virus Disease)) OR (COVID 19 Virus Disease)) OR (COVID-19 Virus Diseases)) OR (Disease, COVID-19 Virus)) OR (Virus Disease, COVID-19)) OR (2019-nCoV Infection)) OR (2019 nCoV Infection)) OR (2019-nCoV Infections)) OR (Infection, 2019-nCoV)) OR (COVID19)) OR (COVID-19 Pandemic)) OR (COVID 19 Pandemic)) OR (Pandemic, COVID-19)) OR (COVID-19 Pandemics)) AND ("Deep Learning"[Mesh] OR ((Learning, Deep) OR (Hierarchical Learning)) OR (Learning, Hierarchical)) AND ((("Severity of Illness Index"[Mesh] OR "Patient Acuity"[Mesh]) OR ((((((((Illness Index Severities) OR (Illness Index Severity)) OR (Disease Severity)) OR (Disease Severities)) OR (Severity, Disease)) OR (Acuity, Patient)) OR (Severity of Illness)) OR (Illness Severity)) OR (Severity)) OR ("Triage"[Mesh] OR Triages))  **Scopus**  TITLE-ABS-KEY ( "COVID-19" OR "COVID 19" OR "SARS-CoV-2 Infection" OR "Infection, SARS-CoV-2" OR "SARS CoV 2 Infection" OR "SARS-CoV-2 Infections" OR "2019 Novel Coronavirus Disease" OR "2019 Novel Coronavirus Infection" OR "2019-nCoV Disease" OR "2019 nCoV Disease" OR "2019-nCoV Diseases" OR "Disease, 2019-nCoV" OR "COVID-19 Virus Infection" OR "COVID 19 Virus Infection" OR "COVID-19 Virus Infections" OR "Infection, COVID-19 Virus" OR "Virus Infection, COVID-19" OR "Coronavirus Disease 2019" OR "Disease 2019, Coronavirus" OR "Coronavirus Disease-19" OR "Coronavirus Disease 19" OR "Severe Acute Respiratory Syndrome Coronavirus 2 Infection" OR "SARS Coronavirus 2 Infection" OR "COVID-19 Virus Disease" OR "COVID 19 Virus Disease" OR "COVID-19 Virus Diseases" OR "Disease, COVID-19 Virus" OR "Virus Disease, COVID-19" OR "2019-nCoV Infection" OR "2019 nCoV Infection" OR "2019-nCoV Infections" OR "Infection, 2019-nCoV" OR "COVID19" OR "COVID-19 Pandemic" OR "COVID 19 Pandemic" OR "Pandemic, COVID-19" OR "COVID-19 Pandemics" ) AND TITLE-ABS-KEY ( "Deep Learning" OR "Learning, Deep" OR "Hierarchical Learning" OR "Learning, Hierarchical" ) AND TITLE-ABS-KEY ( "Severity of Illness Index" OR "Patient Acuity" OR "Illness Index Severities" OR "Illness Index Severity" OR "Disease Severity" OR "Disease Severities" OR "Severity, Disease" OR "Acuity, Patient" OR "Severity of Illness" OR "Illness Severity" OR "Severity" OR "Triage" OR "Triages" )  **LitCovid**  ("Deep Learning" OR “Learning, Deep” OR “Hierarchical Learning” OR “Learning, Hierarchical”) AND ("Severity of Illness Index" OR "Patient Acuity" OR “Illness Index Severities” OR “Illness Index Severity” OR “Disease Severity” OR “Disease Severities” OR “Severity, Disease” OR “Acuity, Patient” OR “Severity of Illness” OR “Illness Severity” OR “Severity” OR "Triage" OR “Triages”)  **Embase**  1 coronavirus disease 2019/ 209509  2 deep learning/ 23764  3 COVID-19 Severity score/ or disease severity assessment/ 7856  4 emergency health service/ 109873  5 3 or 4 117634  6 1 and 2 and 5 40  **Ovid medline**  1 COVID-19/ 157672  2 Deep Learning/ 10837  3 patient acuity/ or "severity of illness index"/ 269426  4 Triage/ 14147  5 3 or 4 282771  6 1 and 2 and 5 21  **Cochran Library**  #1 MeSH descriptor: [COVID-19] explode all trees 1722  #2 (COVID 19):ti,ab,kw OR (SARS-CoV-2 Infection):ti,ab,kw OR (Infection, SARS-CoV-2):ti,ab,kw OR (SARS CoV 2 Infection):ti,ab,kw OR (SARS-CoV-2 Infections):ti,ab,kw OR (2019 Novel Coronavirus Disease):ti,ab,kw OR (2019 Novel Coronavirus Infection):ti,ab,kw OR (2019 nCoV Disease):ti,ab,kw OR (COVID-19 Virus Infection):ti,ab,kw OR (COVID 19 Virus Infection):ti,ab,kw OR (COVID-19 Virus Infections):ti,ab,kw OR (Infection, COVID-19 Virus):ti,ab,kw OR (Virus Infection, COVID-19):ti,ab,kw OR (Coronavirus Disease 2019):ti,ab,kw OR (Disease 2019, Coronavirus):ti,ab,kw OR (Coronavirus Disease-19):ti,ab,kw OR (Coronavirus Disease 19):ti,ab,kw OR (Severe Acute Respiratory Syndrome Coronavirus 2 Infection):ti,ab,kw OR (SARS Coronavirus 2 Infection):ti,ab,kw OR (COVID-19 Virus Disease):ti,ab,kw OR (COVID 19 Virus Disease):ti,ab,kw OR (COVID-19 Virus Diseases):ti,ab,kw OR (Disease, COVID-19 Virus):ti,ab,kw OR (Virus Disease, COVID-19):ti,ab,kw OR (2019 nCoV Infection):ti,ab,kw OR (COVID19):ti,ab,kw OR (COVID-19 Pandemic):ti,ab,kw OR (COVID 19 Pandemic):ti,ab,kw OR (Pandemic, COVID-19):ti,ab,kw OR (COVID-19 Pandemics):ti,ab,kw 10553  #3 MeSH descriptor: [Deep Learning] explode all trees 49  #4 (Learning, Deep):ti,ab,kw OR (Hierarchical Learning):ti,ab,kw OR (Learning, Hierarchical):ti,ab,kw (Word variations have been searched) 1331  #5 MeSH descriptor: [Patient Acuity] explode all trees 21312  #6 (Acuity, Patient):ti,ab,kw OR (Severity of Illness):ti,ab,kw OR (Illness Severity):ti,ab,kw (Word variations have been searched) 46125  #7 MeSH descriptor: [Severity of Illness Index] explode all trees 21161  #8 (Illness Index Severities):ti,ab,kw OR (Illness Index Severity):ti,ab,kw OR (Disease Severity):ti,ab,kw OR (Disease Severities):ti,ab,kw OR (Severity, Disease):ti,ab,kw (Word variations have been searched) 121584  #9 MeSH descriptor: [Triage] explode all trees 322  #10 (Triages):ti,ab,kw (Word variations have been searched) 2015  #11 #5 OR #6 46827  #12 #7 OR #8 122260  #13 #9 OR #10 2015  #14 #11 OR #12 OR #13 140520  #15 #1 OR #2 10553  #16 #3 OR #4 1331  #17 #14 AND #15 AND #16 6 |
| --- |

**Textbox S2. Code of the modules.**

| **MIDAS**  midas tp fp fn tn,res(all)  midas tp fp fn tn,pubbias  midas tp fp fn tn,id(author) ms(0.75) ford fors bfor(dss)  midas tp fp fn tn,plot sroc(both)  midas tp fp fn tn,fagan(0.27)  midas tp fp fn tn,fagan(0.35)  midas tp fp fn tn,lrmat  midas tp fp fn tn, reg(partition source method model parameter image)  **METAPROP**  metaprop events1 total1, random cimethod(exact) lcols(author events1 total1) xlab(0.4, 0.5, 0.6, 0.7, 0.8, 0.9, 1.0) by(partition) dp(2) xtitle(SENSITIVITY,size(3))  metaprop events1 total1, random cimethod(exact) lcols(author events1 total1) xlab(0.4, 0.5, 0.6, 0.7, 0.8, 0.9, 1.0) by(sources) dp(2) xtitle(SENSITIVITY,size(3))  metaprop events1 total1, random cimethod(exact) lcols(author events1 total1) xlab(0.4, 0.5, 0.6, 0.7, 0.8, 0.9, 1.0) by(method) dp(2) xtitle(SENSITIVITY,size(3))  metaprop events1 total1, random cimethod(exact) lcols(author events1 total1) xlab(0.4, 0.5, 0.6, 0.7, 0.8, 0.9, 1.0) by(model) dp(2) xtitle(SENSITIVITY,size(3))  metaprop events1 total1, random cimethod(exact) lcols(author events1 total1) xlab(0.4, 0.5, 0.6, 0.7, 0.8, 0.9, 1.0) by(parameter) dp(2) xtitle(SENSITIVITY,size(3))  metaprop events1 total1, random cimethod(exact) lcols(author events1 total1) xlab(0.4, 0.5, 0.6, 0.7, 0.8, 0.9, 1.0) by(image) dp(2) xtitle(SENSITIVITY,size(3))  metaprop events2 total2, random ftt cimethod(exact) lcols(author events2 total2) xlab(0.4, 0.5, 0.6, 0.7, 0.8, 0.9, 1.0) by(partition) dp(2) xtitle(SPECIFICITY,size(3))  metaprop events2 total2, random cimethod(exact) lcols(author events2 total2) xlab(0.4, 0.5, 0.6, 0.7, 0.8, 0.9, 1.0) by(partition) dp(2) xtitle(SPECIFICITY,size(3))  metaprop events2 total2, random ftt cimethod(exact) lcols(author events2 total2) xlab(0.4, 0.5, 0.6, 0.7, 0.8, 0.9, 1.0) by(sources) dp(2) xtitle(SPECIFICITY,size(3))  metaprop events2 total2, random cimethod(exact) lcols(author events2 total2) xlab(0.4, 0.5, 0.6, 0.7, 0.8, 0.9, 1.0) by(sources) dp(2) xtitle(SPECIFICITY,size(3))  metaprop events2 total2, random ftt cimethod(exact) lcols(author events2 total2) xlab(0.4, 0.5, 0.6, 0.7, 0.8, 0.9, 1.0) by(method) dp(2) xtitle(SPECIFICITY,size(3))  metaprop events2 total2, random cimethod(exact) lcols(author events2 total2) xlab(0.4, 0.5, 0.6, 0.7, 0.8, 0.9, 1.0) by(method) dp(2) xtitle(SPECIFICITY,size(3))  metaprop events2 total2, random ftt cimethod(exact) lcols(author events2 total2) xlab(0.4, 0.5, 0.6, 0.7, 0.8, 0.9, 1.0) by(model) dp(2) xtitle(SPECIFICITY,size(3))  metaprop events2 total2, random cimethod(exact) lcols(author events2 total2) xlab(0.4, 0.5, 0.6, 0.7, 0.8, 0.9, 1.0) by(model) dp(2) xtitle(SPECIFICITY,size(3))  metaprop events2 total2, random ftt cimethod(exact) lcols(author events2 total2) xlab(0.4, 0.5, 0.6, 0.7, 0.8, 0.9, 1.0) by(parameter) dp(2) xtitle(SPECIFICITY,size(3))  metaprop events2 total2, random cimethod(exact) lcols(author events2 total2) xlab(0.4, 0.5, 0.6, 0.7, 0.8, 0.9, 1.0) by(parameter) dp(2) xtitle(SPECIFICITY,size(3))  metaprop events2 total2, random ftt cimethod(exact) lcols(author events2 total2) xlab(0.4, 0.5, 0.6, 0.7, 0.8, 0.9, 1.0) by(image) dp(2) xtitle(SPECIFICITY,size(3))  metaprop events2 total2, random cimethod(exact) lcols(author events2 total2) xlab(0.4, 0.5, 0.6, 0.7, 0.8, 0.9, 1.0) by(image) dp(2) xtitle(SPECIFICITY,size(3)) |
| --- |

| **Table S1.** DTA estimated from all included studies using the (2 × 2) truth table. | | | | | | | | | | | | | | | | | | | | | | | | | | | |  |
| --- | --- | --- | --- | --- | --- | --- | --- | --- | --- | --- | --- | --- | --- | --- | --- | --- | --- | --- | --- | --- | --- | --- | --- | --- | --- | --- | --- | --- |
| Study | Country | Data partition | Data source | No. of patients  (severe) | | AUC | | TP | | FN | | TN | | FP | | ACC | | PRE | | LR+ | | LR- | | DOR | | F1- Score | |  |
| **Assessment** | | | | | | | | | | | | | | | | | | | | | | | | | | | |  |
| Cai et al.2020[28] | China | External test | In the First Affiliated Hospital at Zhejiang University School of Medicine, from January 19 to February 19, 2020 | | 99 (74) | | 0.93 | | 67 | | 7 | | 20 | | 5 | | 0.8788 | | 0.9306 | | 4.5270 | | 0.1182 | | 38.2857 | | 0.9178 | |
| Carvalho et al.2020[39] | Brazil | Training | In three hospitals of Brazil, from April to June 2020 | 469(184) | | 0.99 | | 167 | | 17 | | 265 | | 11 | | 0.9391 | | 0.9382 | | 22.7727 | | 0.0962 | | 236.6578 | | 0.9227 | |  |
|  |  | Validation |  | 99(40) | | 0.94 | | 38 | | 2 | | 56 | | 3 | | 0.9495 | | 0.9268 | | 18.6833 | | 0.0527 | | 354.6667 | | 0.9383 | |  |
|  |  | Internal test |  | 97(35) | | 0.90 | | 31 | | 4 | | 61 | | 1 | | 0.9485 | | 0.9688 | | 54.9143 | | 0.1162 | | 472.7500 | | 0.9254 | |  |
| Li et al.2020[29] | China | Training | Another multi-center pneumonia dataset | NA | | NA | | NA | | NA | | NA | | NA | | NA | | NA | | NA | | NA | | NA | | NA | |  |
|  |  | External test | In the First Hospital of Changsha, from January 23 to February 12, 2020 | 196 (32) | | 0.97 | | 30 | | 2 | | 144 | | 20 | | 0.8878 | | 0.6000 | | 7.6875 | | 0.0712 | | 108.0000 | | 0.7317 | |  |
| Xiao et al.2020[37] | China | Training | In the People’s Hospital of Honghu, from January 1 to March 18, 2020 | 303 (48) | | 0.99 | | 46 | | 2 | | 249 | | 6 | | 0.9736 | | 0.8846 | | 40.7292 | | 0.0427 | | 954.5000 | | 0.9200 | |  |
|  |  | External test | In the First Affiliated Hospital of Nanchang University, from January 1 to March 18, 2020 | 105 (40) | | 0.89 | | 35 | | 5 | | 51 | | 14 | | 0.8190 | | 0.7143 | | 4.0625 | | 0.1593 | | 25.5000 | | 0.7865 | |  |
| Yu et al.2020[30] | China | Training | In the First Affiliated Hospital of Bengbu Medical college, the First Affiliated Hospital of Anhui Medical University and Fuyang Second People’s Hospital, from January 24 to February 12, 2020 | 162 (28) | | NA | | NA | | NA | | NA | | NA | | NA | | NA | | NA | | NA | | NA | | NA | |  |
|  |  | Internal test |  | 40 (13) | | 0.99 | | 12 | | 1 | | 26 | | 1 | | 0.9500 | | 0.9231 | | 24.9231 | | 0.0799 | | 312.0000 | | 0.9231 | |  |
| Aboutalebi et al.2021[40] | America, Brazil, Canada and Turkey | Internal test | In Koç University Hospital, Turkey, UC San Francisco, USA, Unity Health Toronto, Canada, and Universidade de São Paulo, Brazil. | 150 (98) | | 0.96 | | 91 | | 7 | | 48 | | 4 | | 0.9267 | | 0.9579 | | 12.0714 | | 0.0774 | | 156.0000 | | 0.9430 | |  |
| Feng et al.2021[38] | China | Training | In the Third Xiangya Hospital, First Hospital of Changsha, First Hospital of Yueyang, Second Hospital of Changde and Central Hospital of Xiangtan, from January 17 to February 17, 2020 | 424(37) | | 0.96 | | 28 | | 9 | | 373 | | 14 | | 0.9458 | | 0.6667 | | 20.9189 | | 0.2524 | | 82.8889 | | 0.7089 | |  |
|  |  | External test | In Central Hospital of Shaoyang and Central Hospital of Loudi, from January 17 to February 17, 2020 | 98(8) | | 0.97 | | 8 | | 1 | | 77 | | 13 | | 0.8586 | | 0.3810 | | 6.1538 | | 0.1299 | | 47.3846 | | 0.5333 | |  |
| He et al.2021[31] | China | Internal test | In seven hospitals of China | 191(51) | | 0.99 | | 49 | | 2 | | 190 | | 1 | | 0.9876 | | 0.9800 | | 183.5098 | | 0.0394 | | 4655.0000 | | 0.9703 | |  |
| Ho et al.2021[41] | Republic of Korea | Training | Five hospitals in Daegu, South Korea, from January 31 to April 10, 2020 | 239 (35) | | 0.92 | | 28 | | 7 | | 198 | | 6 | | 0.9456 | | 0.8235 | | 27.2000 | | 0.2061 | | 132.0000 | | 0.8116 | |  |
|  |  | Internal test |  | 58 (7) | | 0.92 | | 6 | | 1 | | 49 | | 2 | | 0.9483 | | 0.7500 | | 21.8571 | | 0.1487 | | 147.0000 | | 0.8000 | |  |
| Li et al.2021[32] | China | Internal test | In the Second Xiangya Hospital of Central South University and its collaborating hospitals | 229 (50) | | 0.98 | | 47 | | 3 | | 173 | | 6 | | 0.9607 | | 0.8868 | | 28.0433 | | 0.0621 | | 451.7222 | | 0.9126 | |  |
| Udristoiu et al.2021[42] | Italy | Training | In Victor Babes University Hospital in Craiova, Romania, from September 2020 to May 2021 | 380 (139) | | NA | | NA | | NA | | NA | | NA | | NA | | NA | | NA | | NA | | NA | | NA | |  |
|  |  | Internal test |  | 95 (35) | | 0.98 | | 34 | | 1 | | 60 | | 0 | | 0.9895 | | 1.0000 | | NA | | 0.0286 | | NA | | 0.9855 | |  |
| Ortiz et al.2022[43] | Algeria | Internal test | In Hakim Saidane Biskra Hospital and Ziouch Mohamed Tolga Hospital, from June to December 2020 | 596 (107) | | NA | | 95 | | 12 | | 470 | | 19 | | 0.9480 | | 0.8333 | | 22.8505 | | 0.1167 | | 195.8333 | | 0.8597 | |  |
| **Prediction** | | | | | | | | | | | | | | | | | | | | | | | | | | | |  |
| Ning et al.2020[33] | China | Training | In Union Hospital, from November 14 to 30, 2019 and from January 25 to February 20, 2020 | 649 (211) | | 0.93 | | 150 | | 61 | | 406 | | 32 | | 0.8567 | | 0.8242 | | 9.7305 | | 0.3119 | | 31.1988 | | 0.7634 | |  |
|  |  | External test | In Liyuan Hospital, from January 25 to February 20, 2020 | 252 (63) | | 0.88 | | 50 | | 13 | | 148 | | 41 | | 0.7857 | | 0.5495 | | 3.6585 | | 0.2635 | | 13.8837 | | 0.6494 | |  |
| Xiao et al.2020[37] | China | Training | In the People’s Hospital of Honghu, from January 1 to March 18, 2020 | | 255(12) | | 0.96 | | 10 | | 2 | | 237 | | 6 | | 0.9686 | | 0.6250 | | 33.7500 | | 0.1709 | | 197.5000 | | 0.7143 | |
|  |  | External test | In the First Affiliated Hospital of Nanchang University, from January 1 to March 18, 2020 | 65 (11) | | 0.92 | | 9 | | 2 | | 42 | | 12 | | 0.7846 | | 0.4285 | | 3.6818 | | 0.2338 | | 15.7500 | | 0.5625 | |  |
| Zhang et al.2020[44] | China | Training | In Sun Yat-sen Memorial Hospital, Third Affiliated Hospital of Sun Yat-sen University, the first Affiliated Hospital of Anhui Medical University, West China Hospital, Nanjing Renmin Hospital, Yichang Central People’s Hospital and Renmin Hospital of Wuhan University | 843 (NA) | | NA | | NA | | NA | | NA | | NA | | NA | | NA | | NA | | NA | | NA | | NA | |  |
|  |  | Internal test |  | 432 (158) | | 0.91 | | 126 | | 32 | | 238 | | 36 | | 0.8426 | | 0.7778 | | 6.0696 | | 0.2332 | | 26.0313 | | 0.7875 | |  |
| Fang et al.2021[34] | China | Training | In Tongji Hospital (the Zhongfa branch), from January 13 to March 16, 2020 | 435 (81) | | NA | | NA | | NA | | NA | | NA | | NA | | NA | | NA | | NA | | NA | | NA | |  |
|  |  | Validation |  | 109 (47) | | 0.92 | | 42 | | 5 | | 54 | | 8 | | 0.8807 | | 0.8400 | | 6.9255 | | 0.1221 | | 56.7000 | | 0.8660 | |  |
|  |  | Internal test | In Tongji Hospital (Guanggu branch), from January 13 to March 16, 2020 | 363 (154) | | 0.89 | | 117 | | 37 | | 175 | | 34 | | 0.8044 | | 0.7748 | | 4.6702 | | 0.2869 | | 16.2758 | | 0.7672 | |  |
|  |  | External test | In Wuhan Pulmonary Hospital, from January 3 to February 13, 2020 | 133 (54) | | 0.86 | | 40 | | 14 | | 68 | | 11 | | 0.8120 | | 0.7843 | | 5.3199 | | 0.3012 | | 17.6623 | | 0.7619 | |  |
| Feng et al.2021[38] | China | External test | In Central Hospital of Shaoyang and Central Hospital of Loudi, from January 17 to February 17, 2020 | 98 (8) | | 0.88 | | 6 | | 2 | | 79 | | 11 | | 0.8673 | | 0.3529 | | 6.1364 | | 0.2848 | | 21.5455 | | 0.4800 | |  |
| Jiao et al.2021[45] | America | Training | In the University of Pennsylvania Health System in Philadelphia, from March 9 to July 20, 2020 | 1285 (300) | | NA | | NA | | NA | | NA | | NA | | NA | | NA | | NA | | NA | | NA | | NA | |  |
|  |  | Validation |  | 183 (41) | | NA | | NA | | NA | | NA | | NA | | NA | | NA | | NA | | NA | | NA | | NA | |  |
|  |  | Internal test |  | 366 (84) | | 0·85 | | 62 | | 22 | | 241 | | 41 | | 0.8279 | | 0.6019 | | 5.0767 | | 0.3065 | | 16.5654 | | 0.6631 | |  |
|  |  | External test | In Brown University affiliated hospitals in Providence, from March 1 to July 18, 2020 | 475 (125) | | 0.79 | | 91 | | 34 | | 245 | | 105 | | 0.7074 | | 0.4643 | | 2.4267 | | 0.3886 | | 6.2451 | | 0.5670 | |  |
| Kwon et al.2021[46] | America | Training | In three hospitals in New York City, from March 10 to 29, 2020 | 283 (111) | | NA | | NA | | NA | | NA | | NA | | NA | | NA | | NA | | NA | | NA | | NA | |  |
|  |  | Validation |  | 55 (27) | | NA | | NA | | NA | | NA | | NA | | NA | | NA | | NA | | NA | | NA | | NA | |  |
|  |  | Internal test |  | 156 (46) | | 0.88 | | 38 | | 8 | | 78 | | 32 | | 0.7436 | | 0.5426 | | 2.8397 | | 0.2453 | | 11.5781 | | 0.6552 | |  |
| Lassau et al.2021[47] | France | Training | In Kremlin-Bicêtre, Paris, from February 2 to March 20, 2020 | 646 (NA) | | NA | | NA | | NA | | NA | | NA | | NA | | NA | | NA | | NA | | NA | | NA | |  |
|  |  | Internal test |  | 150 (44) | | 0.76 | | 31 | | 13 | | 80 | | 26 | | 0.7400 | | 0.5439 | | 2.8724 | | 0.3915 | | 7.3373 | | 0.6139 | |  |
| Shi et al.2021[35] | China | Internal test | In the Centre for Disease Control, Shanghai, from January 20 to February 10, 2020 | 196 (45) | | 0.90 | | 36 | | 9 | | 130 | | 21 | | 0.8469 | | 0.6316 | | 5.7524 | | 0.2323 | | 24.7619 | | 0.7059 | |  |
| Soda et al.2021[48] | Italy | Internal test | In six Italian hospitals of Italy, from March to June 2020 | 820 (436) | | NA | | 325 | | 111 | | 288 | | 96 | | 0.7476 | | 0.7720 | | 2.9817 | | 0.3394 | | 8.7838 | | 0.7585 | |  |
| Chieregato et al.2022[49] | Italy | Training | In Fondazione Poliambulanza Istituto Ospedaliero, Brescia, from February 20 to May 6, 2020 | 451 (147) | | 0.96 | | NA | | NA | | NA | | NA | | NA | | NA | | NA | | NA | | NA | | NA | |  |
|  |  | Internal test |  | 107 (31) | | 0.95 | | 26 | | 5 | | 71 | | 5 | | 0.9065 | | 0.8387 | | 12.7484 | | 0.1726 | | 73.8400 | | 0.8387 | |  |
| Chen et al.2022[36] | China | Internal test | In Tianyou Hospital, which is Affiliated with Wuhan University of Science and Technology, the First Affiliated Hospital of Zhejiang University School of Medicine, and the First hospital of Jiaxing | 140 (70) | | 0.76 | | 55 | | 15 | | 51 | | 19 | | 0.7571 | | 0.7432 | | 2.8947 | | 0.2941 | | 9.8421 | | 0.7639 | |  |
| Wang et al.2022[50] | America and China | Training | In Xiangya Hospital, Yongzhou Central Hospital, Changde Second People’s Hospital, Affiliated Nan Hua Hospital, Loudi Central Hospital, Chenzhou Second People’s Hospital, Zhuzhou Central Hospital, Yiyang City Central Hospital, The First Hospital of Changsha, Hospital of the University of Pennsylvania and Rhode Island Hospital | 737 (NA) | | NA | | NA | | NA | | NA | | NA | | NA | | NA | | NA | | NA | | NA | | NA | |  |
|  |  | Validation |  | 105 (NA) | | NA | | NA | | NA | | NA | | NA | | NA | | NA | | NA | | NA | | NA | | NA | |  |
|  |  | Internal test |  | 209 (45) | | 0.86 | | 33 | | 12 | | 146 | | 18 | | 0.8565 | | 0.6471 | | 6.6815 | | 0.2995 | | 22.3056 | | 0.6875 | |  |

DTA diagnostic test accuracy, AUC area under curve, TP true positive, FN false negative, TN true negative, FP false positive, ACC accuracy, PRE precision, LR+ positive likelihood ratio, LR- negative likelihood ratio, NA not available

| **Table S2. Detailed characteristics of the studies.** | | | | | | | | |
| --- | --- | --- | --- | --- | --- | --- | --- | --- |
| Study | Type of Research/ Training method | Data  sources | Deep learning model networks | Training epochs/ Learning rate/ Batch  size | Threshold for the Classifiers (C) or Predictions (P) | Optimizer/ Validation strategies/ Interpretability | Criteria for severe patients | Parameter used by the model |
| Cai et al.2020 | Retrospective/ Pre-trained | Single/ benchmark | UNet | 10/ 1e-4/ 8 | C: RF models +  Boruta algorithm | Mini-batch + Adam/ Cross-validation (10-fold, 100 repetitions)/ NA | RR ≥ 30 breaths/min, SaO_2_ ≤ 93% in a resting state, PaO_2_/ FiO_2_ ≤ 300mmHg, Significant progression of pulmonary lesions (over 50%) within 24–48 h, Mechanical ventilation, ICU admission or Shock | Age, Image parameters (CT), Lymphocyte count, Neutrophil count, PaO_2_, SaO_2_ |
| Carvalho et al.2020 | Retrospective/ Pre-trained | Multiple/ benchmark | ANN | 6/ NA/ NA | C: ANN classifier | NA/ Cohort validation/ Quantitative results | PI ≥ 3 zone score | Image parameters (Chest CT) |
| Li et al. 2020 | Retrospective/ Customized | Single/ benchmark | UNet + ResNet-34 | NA/ NA/ NA | C: AI classification  model | NA/ NA/ NA | RR ≥ 30 breaths/min, SaO2 ≤ 93% in a resting state, PaO2/ FiO2 ≤ 300mmHg, Significant progression of pulmonary lesions (over 50%) within 24–48 h, Respiratory failure with the requirement of mechanical ventilation, ICU admission, Shock or Multiple organ failure | Image parameters (Chest CT) |
| Ning et al.2020 | Retrospective/ Pre-trained | Multiple/ benchmark | InceptionV3 + DenseNet-121 + VGG-16 | 500/ 5e-2/ 64 | C: HUST-19, P: Dropout | Adam/ Cross-validation (10-fold, 10 repetitions)/ NA | RR ≥ 30 breaths/min, SaO2 ≤ 93% in a resting state, PaO2/ FiO2 ≤ 300mmHg, Significant progression of pulmonary lesions (over 50%) within 24–48 h, Respiratory failure with the requirement of mechanical ventilation, ICU admission, Shock or Multiple organ failure | Age, Albumin, ALT, AST, Brain natriuretic peptide, CD4^+^ T cell, Calcium, Creatinine, CRP, Eosinophil count, Globulin, γ-Glutamyl transpeptidase, Image parameters (Chest CT), Lymphocyte count, Monocyte count, Neutrophil count, Platelet, Procalcitonin, Sex, Sodium, Total bilirubin, Urea, WBC count |
| Xiao et al. 2020 | Retrospective/ Pre-trained | Multiple/ benchmark | ResNet-34 | 100/ NA/ NA | C: Patient-level CT classification,  P: MIL | NA/ Cross-validation (5-fold)/ NA | RR ≥ 30 breaths/min, SaO2 ≤ 93% in a resting state, PaO2/ FiO2 ≤ 300mmHg, Significant progression of pulmonary lesions (over 50%) within 24–48 h, Respiratory failure with the requirement of mechanical ventilation, ICU admission, Shock or Multiple organ failure | Image parameters (Initial Chest CT) |
| Yu et al. 2020 | Retrospective/ Pre-trained | Multiple/ benchmark | DenseNet-201 | NA/ NA/ NA | C: Cubic SVM | NA/ Cross-validation (10-fold)/ NA | RR ≥ 30 breaths/min, SaO2 ≤ 93% in a resting state, PaO2/ FiO2 ≤ 300mmHg, Significant progression of pulmonary lesions (over 50%) within 24–48 h, Respiratory failure with the requirement of mechanical ventilation, ICU admission, Shock or Multiple organ failure | Image parameters (Chest CT) |
| Zhang et al.2020 | Retrospective/ Customized | Multiple/ benchmark | UNet + FCN + DeepLabv3 + ResNet-18 | 20/ 1e-5/ 8 | C: Softmax + GBDT, P: LightGBM + CoxPH | SGD + Adam/ Cross-validation (5-fold)/ SHAP | ICU admission, the use of mechanical ventilation, or death | Age, Albumin, CRP, Image parameters (Chest CT), SaO_2_ |
| Aboutalebi et al.2021 | Retrospective/ Customized | Multiple/ benchmark | COVIDNet | 30/ 1e-4/ 32 | C: COVID-Net CXR-S | Adam/ Radiologist validation/ GSInquire | Opacities in 3 or more lung zones | Image parameters (Chest X-ray) |
| Fang et al. 2021 | Retrospective/ Pre-trained | Multiple/ benchmark | 3D ResNet | 100/ 5e-2/20 | C: Softmax + MLP,  P: LSTM | Adam/ Cross-validation (5-fold)/ Grad-CAM | RR ≥ 30 breaths/min, SaO2 ≤ 93% in a resting state, PaO2/ FiO2 ≤ 300mmHg, Significant progression of pulmonary lesions (over 50%) within 24–48 h, Respiratory failure with the requirement of mechanical ventilation, ICU admission, Shock or Multiple organ failure | Albumin, AST, Brain natriuretic peptide, CD4^+^T cells count, CRP, Creatinine, Fever, Hypertension, Image parameters (Chest CT), Troponin , WBC count, γ-Glutamyl transpeptidase |
| Feng et al.2021 | Retrospective/ Customized | Multiple/ benchmark | UNet++ | NA/ NA/ NA | C: XGBoost | Grid search/ Cross-validation (5-fold)/ NA | RR ≥ 30 breaths/min, SaO2 ≤ 93% in a resting state, PaO2/ FiO2 ≤ 300mmHg, Significant progression of pulmonary lesions (over 50%) within 24–48 h, Respiratory failure with the requirement of mechanical ventilation, ICU admission, Shock or Multiple organ failure | Cardiovascular or cerebrovascular diseases, COPD, Diabetes, Hs-cTnI, Hypertension, Image parameters (Chest CT), LDH |
| He et al.2021 | Retrospective/ Customized | Multiple/ benchmark | UNet | 100/ 1e-2/ NA | C: M^2^UNet + GCP | SGD/ Cross-validation (5-fold)/ NA | RR ≥ 30 breaths/min, SaO2 ≤ 93% in a resting state, PaO2/ FiO2 ≤ 300mmHg, Significant progression of pulmonary lesions (over 50%) within 24–48 h, Respiratory failure with the requirement of mechanical ventilation, ICU admission, Shock or Multiple organ failure | Image parameters (3D Chest CT) |
| Ho et al. 2021 | Retrospective/ Pre-trained | Multiple/ benchmark | ResNet-50 + InceptionV3 + DenseNet121 + ANN | 3000/ 1e-3/ NA | C: Sigmoid | Adam + Binary cross-entropy/ Cross-validation (5-fold)/ Grad-CAM | High-flow nasal cannula, mechanical ventilator care, septic shock, acute kidney injury, continuous renal replacement therapy, extracorporeal membrane oxygenation, ICU admission or death | CRP, Image parameters (3D CT), SaO_2_, Respiratory rate, Systolic blood pressure, WBC count |
| Jiao et al. 2021 | Retrospective/ Pre-trained | Multiple/ benchmark | UNet + VGG-11 + EfficientNet-B0 | NA/ 5e-4/ NA | C: Softmax,  P: Progression prediction model | NA/ Cohort validation/ NA | Utilisation of mechanical ventilation, ICU admission, or death | Age, Cardiovascular Disease, Chronic Kidney Disease, Chronic Liver Disease, COPD, Creatinine, CRP, Diabetes, Fever, Hypertension, Image parameters (Chest X-ray), Lymphocyte count, Malignant Tumor, Sex, SpO_2_, WBC count |
| Kwon et al. 2021 | Retrospective/ Pre-trained | Multiple/ benchmark | DenseNet-121 | 10/ 1e-5/ NA | C: Sigmoid,  P: DL model | Adam + Binary cross-entropy/ Cohort validation/ NA | admission, intubation, or death | Image parameters (Chest X-ray) |
| Lassau et al.2021 | Retrospective/ Pre-trained | Multiple/ benchmark | ResNet50 + EfficientNetB0 + Penalized Logistic regression + UNet | NA/ NA/ NA | C: AI-segment,  P: AI-severity | NA/ Cross-validation (5-fold)/ Logistic regression | oxygen flow rate ≥ 15 L/min, the need for mechanical ventilation or death | Age, Image parameters (Chest CT), Platelet count, SaO_2_, Sex, Urea |
| Li et al.2021 | Retrospective/ Customized | Single/ benchmark | CCN | 50/ 1e-4/ 1 | C: Sigmoid | Adam/ Cross-validation (10-fold)/ Predicted label + Visualization of the attention mechanism | RR ≥ 30 breaths/min, SaO2 ≤ 93% in a resting state, PaO2/ FiO2 ≤ 300mmHg, Significant progression of pulmonary lesions (over 50%) within 24–48 h, Respiratory failure with the requirement of mechanical ventilation, ICU admission, Shock or Multiple organ failure | Image parameters (Chest CT) |
| Shi et al.2021 | Retrospective/ Customized | Single/ benchmark | VNet + LASSO Logistic regression | NA/ NA/ NA | C: uAI-Discover-NCP, P: Nomogram model | NA/ Cross-validation (10-fold)/ NA | RR ≥ 30 breaths/min, SaO2 ≤ 93% in a resting state, PaO2/ FiO2 ≤ 300mmHg, Significant progression of pulmonary lesions (over 50%) within 24–48 h, Respiratory failure with the requirement of mechanical ventilation, ICU admission, Shock or Multiple organ failure | Age, CD4^+^ T cell count, CRP, Image parameters (Chest CT), LDH |
| Soda et al.2021 | Retrospective/ Pre-trained | Multiple/ benchmark | UNet +ResNet-50 | 20/ 1e-4/ 16 | C: Supervised classifier, P: MLP | Adam + SGD/ Cross-validation (10-fold, 20 repetitions)/ NA | non-invasive ventilation support, ICU admission and deceased patients | Age, D-dimer, Diabetes, Image parameters (Chest X-ray), LDH, Sex, SaO2, WBC count |
| Udristoiu et al.2021 | Retrospective/ Customized | Single/ benchmark | VGG-19 + ResNet-50 + DenseNet-121 + InceptionV3 | 100/ 1e-4/ 32 | C: CXR-Score, P: AdaBoost + RF + XGBoost + CatBoost | Adam + RMSprop/ Cross-validation (5-fold)/ Evaluated on the SCB testing dataset | oxygen flow rate, the necessity of mechanical ventilation or patient death | Image parameters (Chest X-ray) |
| Chieregato et al.2022 | Retrospective/ Pre-trained | Single/ benchmark | 3D CNN | 50/ 3e-5/ NA | C: CatBoost classifier, P: BorutaSHAP | Optuna + SGD/ Cross-validation (10-fold)/ SHAP analysis | death or ICU admission | Age, Creatinine, Creatine kinase, Image parameters (Chest CT) |
| Chen et al. 2022 | Retrospective/ Pre-trained | Multiple/ benchmark | Mask R-CNN + ANN | NA/ NA/ NA | C: SelectKBest, P: Severe COVID-19 early warning model | NA/ Cross-validation (10-fold)/ Statistical analysis of clinical data | RR ≥ 30 breaths/min, SaO2 ≤ 93% in a resting state, PaO2/ FiO2 ≤ 300mmHg, Significant progression of pulmonary lesions (over 50%) within 24–48 h, Respiratory failure with the requirement of mechanical ventilation, ICU admission, Shock or Multiple organ failure | ALT, Image parameters (Chest CT), Lymphocyte count, Neutrophil count, PaO2, Platelet count |
| Ortiz et al.2022 | Retrospective/ Customized | Multiple/ benchmark | DenseNet-161 | 10/ 1e-6/ 20 | C: SVM + LDA | RMSprop/ Cross-validation (5-fold)/ NA | the infection in the different CT slices for a patient suffering from COVID-19 | Image parameters (Chest CT) |
| Wang et al. 2022 | Retrospective/ Pre-trained | Multiple/ benchmark | EfficientNet | NA/ NA/ NA | C: Pattern classification, P: Keras + Tensorflow | NA/ Cohort validation/ NA | mechanical ventilation, ICU admission or death | Image parameters (Chest CT), Age, Cancer, Cardiovascular disease, Chronic kidney disease, Chronic liver disease, COPD, Diabetes, Fever, Hypertension, HIV, Lymphocyte count, Sex, WBC count |

ADASYN adaptive synthetic sampling approach for imbalanced learning, ANN artificial neural network, AST aspartate aminotransferase, ALT alanine aminotransferase, BUN blood urea nitrogen, CNN convolutional neural networks, COPD chronic obstructive pulmonary disease, CoxPH Cox proportional-hazards, CRP C-reactive protein, DL deep learning, FCN fully connected neural network, FiO2 fraction of inspired oxygen, GCP global contrast pooling, Grad-CAM Gradient-weighted Class Activation Mapping, ICU intensive care unit, IoU Intersection-over-Union, LDA Latent Dirichlet Allocation, LDH lactate dehydrogenase, LightGBM Light Gradient Boosting Machine, LSTM Long Short-Term Memory, MIL Multiple instance learning, MLP Multilayer Perceptron, NA not available, PaO2 partial pressure of oxygen, PI pulmonary insufficiency, RF random forest, RR respiration rate, SaO2 oxygen saturation, SGD Stochastic Gradient Descent, SVM Support Vector Machine, WBC white blood cell

Image parameters include total lesion volume, volume change, proportion of lesions, mean density, edge clarity, pleural distance, form, mean lesion volume, MOICT, lesion range score, number of segments involved, CT/CXR severity score, consolidation, ground-glass opacification (GGO).

**Figure S1. Deek funnel plot.**


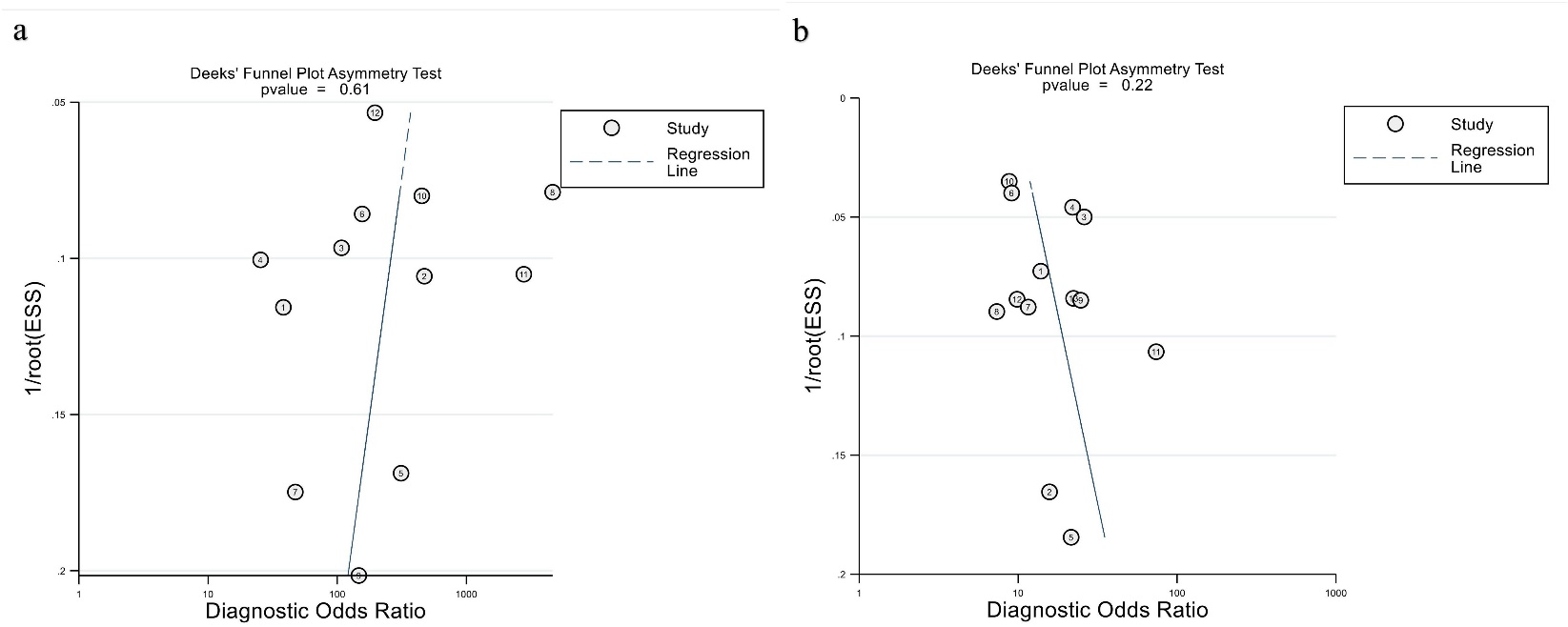


Deek funnel plot asymmetry test for publication bias, with P < 0·1 indicating publication bias. a. The assessed value of DL models for disease severity. b. The predictive value of DL models for disease severity.

**Figure S2. Univariable meta-regression and subgroup analyses.**


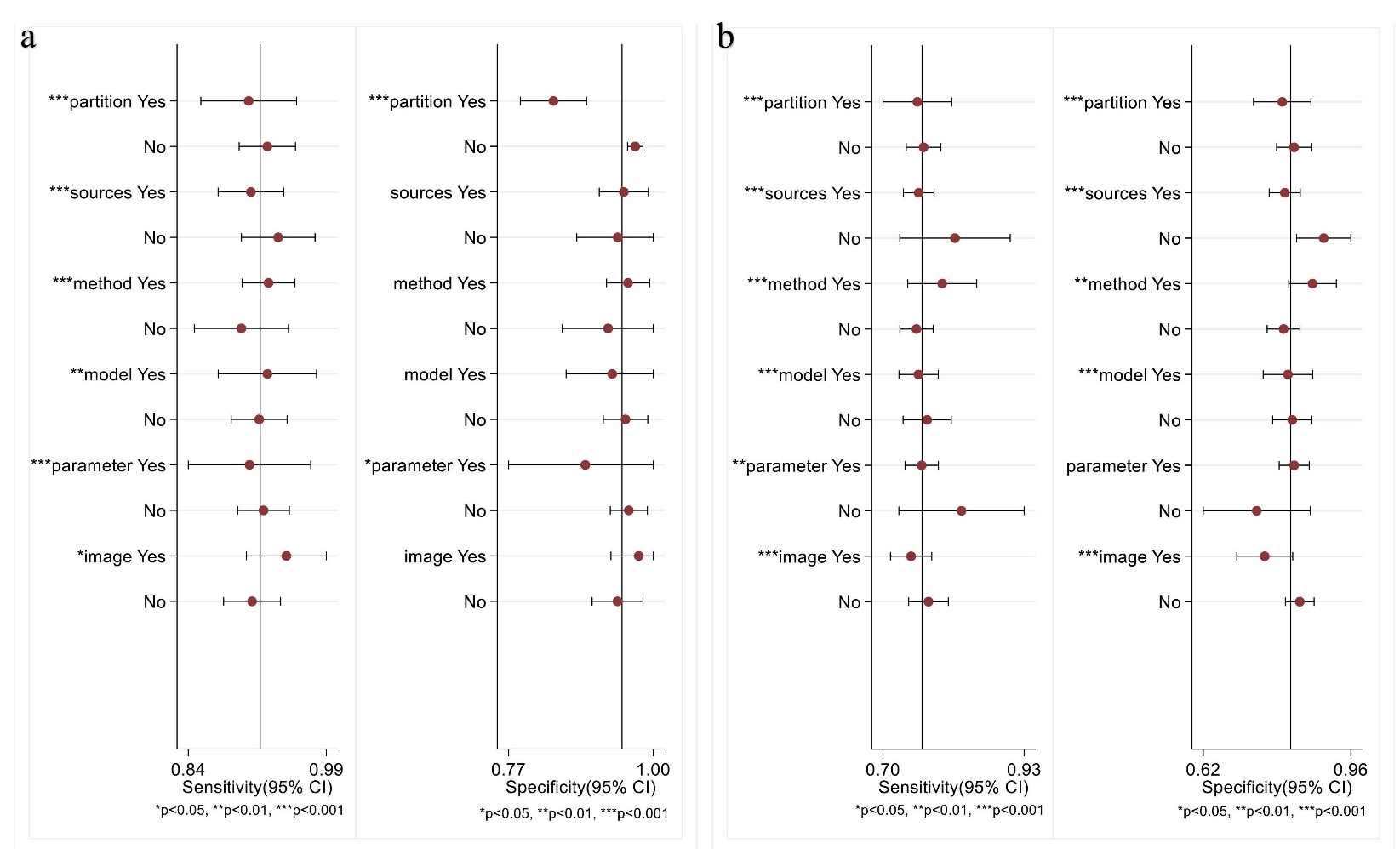


We conducted the subgroup analyses in six areas, including the Data partition (Internal test or External test), the Data sources (Single/ benchmark or Multiple/ benchmark), the Training method (Pre-trained or Customized), the DL model networks (ResNet or Other networks), the Parameter used by the model (Only image parameter or Clinical and image parameter) and the Image (CT or X-ray).

a. The heterogeneity of DL model sensitivities for assessing disease severity was affected by data partition (P <.001), data sources (P <.001), training method (P <.001), DL model networks (P <.01), input parameter (P <.001), and image (P <.05). The heterogeneity of specificity was affected by partition (P <.001), and parameter (P <.05). The heterogeneity of specificity was affected by partition (P <.001), sources (P <.001), training method (P <.01), model networks (P <.001), and image (P <.001). Data partition (P＜.001), sources (P＜.001), training method (P＜.01), DL model networks (P <.01) and parameter (P＜.001) affected the heterogeneity of model sensitivities for assessing disease severity. Heterogeneity of DL model specificity for assessing disease severity was impacted by data partition (P＜.001) and parameter used by the model (P＜.01).

b. The heterogeneity of sensitivities for predicting was affected by partition (P <.001), sources (P <.001), training method (P <.001), model networks (P <.001), parameter (P <.001), and image (P <.001). All six areas exert an effect on the heterogeneity of model sensitivity and specificity for predicting disease severity (P＜.01).

**Figure S3. Subgroup analysis corresponding plots.**

Data partition (Assessment)

Data partition (Prediction)

Data sources (Assessment)

Data sources (Prediction)

Training method (Assessment)

Training method (Prediction)

Deep learning model networks (Assessment)

Deep learning model networks (Prediction)

Input parameter (Assessment)

Input parameter (Prediction)

Image (Assessment)

Image (Prediction)

**Table S3. Predictors affecting the severity of COVID-19.**

| Basic Information | Clinical Tests | Diseases and Complication | Treatments |
| --- | --- | --- | --- |
| **Supported**: BMI,[68] Frailty,[69] Gender,[70] Hyperglycemia,[71] Hypertension,[72] Obesity,[68] Previous influenza vaccination,[73] Repeated positivity,[74] SOAT,[75] Vaccines,[76] VFA.[77]  **Uncertain, controversial, or unsupported:** Age,[78] Ethnicity,[79] Smoking,[80,81] SOT,[82] KTR.[83] | **Supported:** Cardiac injury biomarkers: ApoA1 and ApoB,[84] BNP,[85] CK,[86] CK-MB,[87] LDH,[88] NT-proBNP,[85] TnⅠ;[89] Coagulation parameters: D-D,[90] FIB,[91] INR,[92] MPV,[93] PLT,[91] PT;[94] Cytokines: IL-6,[86] IL-8,[95] IL-10,[96] VWF,[97] VWF-ADAMTS13 axis;[98] Inflammatory markers: CRP,[99] EC,[100] LEC,[101] LYC,[101] NLR;[102] Iron related biomarkers: Ferritin,[103] Serum iron,[103] TIBC;[103] Liver biochemistries: Albumin,[104] AST,[104] Prealbumin,[105] TB;[104] Others: C3 and C4,[106] Cystatin C,[107] fT3,[108] IFITM3,[109] LUS,[110] RDW,[111] SAA,[112] IgG N-glycome.[113]  **Uncertain, controversial, or unsupported:** ALT,[104] ApoB/ApoA1 ratio,[84] APTT,[114] IgA,[115] IL-6/IFN-γ ratio,[116] PCT,[117] TNF-α.[96] | **Supported:** Acute pancreatitis,[118] AKI,[119–121] ALI,[122] Cancer,[123] Cardio-Cerebrovascular disease,[124] CKD,[119] COPD,[125] CRD,[125] CVD,[126,127] Diabetes,[128–132] DIC,[133,134] Dyslipidemia,[135] Encephalitis,[136] Hematological malignancies,[137] HF,[138] Hyponatremia, Hypernatremia, and Hypocalcemia,[139] MAFLD,[140] OSA,[141] Pleural effusion,[142] RVD,[143] SARS-CoV-2 RNAemia,[144] Stroke,[145] VTE;[146] Nervous system diseases:[147] Delirium,[148] Dementia,[149] Epilepsy,[150] Mental disorder.[151]  **Uncertain, controversial, or unsupported:** Asthma,[152] Autoimmune disease.[153] | **Supported:** AC,[154] Insulin,[155] Opioid,[156] Statins.[157]  **Uncertain, controversial, or unsupported:** Angiotensin pathway inhibitors,[158] Antihypertensive drug,[159] Chemotherapy,[160,161] Corticosteroid,[162,163] PPI,[164] RAAS inhibitors, Statin,[165] Tocilizumab,[166] Vitamin C,[167] Vitamin D.[168,169] |

AC anticoagulation, AKI acute kidney injury, ALI acute liver injury, ALT alanine aminotransferase, Apo apoliprotein, APTT activated partial thromboplastin time, AST aspartate aminotransferase, BNP B-type natriuretic peptide, C3 serum complement C3, C4 serum complement C4, CK creatine kinase, CK-MB creatine kinase myocardial band, CKD chronic kidney disease, COPD chronic obstructive pulmonary disease, CRD chronic respiratory disease, CRP C-reactive protein, CVD cardiovascular disease, D-D D-dimer, DIC disseminated intravascular coagulation, EC eosinophil count, FIB fibrinogen, fT3 free triiodothyronine, HF heart failure, IFITM3 interferon-induced transmembrane protein 3 gene, IL interleukin, INR international normalized ratio, KTR kidney transplantation recipient, LDH lactate dehydrogenase, LEC leukocyte count, LUS lung ultrasound, LYC lymphocyte count, MAFLD metabolic associated fatty liver disease, MPV mean platelet volume, NLR neutrophil-to-lymphocyte ratio, NT-proBNP N-terminal proBNP, PCT procalcitonin, PLT platelet count, PPI proton pump inhibitor, PT prothrombin time, RAAS renin-angiotensin aldosterone system, RDW red blood cell distribution width, RVD right ventricular dysfunction, SAA serum amyloid A, SOT solid organ transplantation, SOAT symptom onset to admission time, TB total bilirubin, TIBC total iron banding capacity, TnⅠtroponinⅠ, TNF-α tumor necrosis factor-alpha, VFA visceral fat area, VTE venous thromboembolism, VWF von willebrand factor.

**References**

28 Cai W, Liu T, Xue X, *et al.* CT Quantification and Machine-learning Models for Assessment of Disease Severity and Prognosis of COVID-19 Patients. *Academic Radiology* 2020;**27**:1665–78. doi:10.1016/j.acra.2020.09.004

29 Li Z, Zhong Z, Li Y, *et al.* From community-acquired pneumonia to COVID-19: a deep learning-based method for quantitative analysis of COVID-19 on thick-section CT scans. *Eur Radiol* 2020;**30**:6828–37. doi:10.1007/s00330-020-07042-x

30 Yu Z, Li X, Sun H, *et al.* Rapid identification of COVID-19 severity in CT scans through classification of deep features. *Biomed Eng Online* 2020;**19**:63. doi:10.1186/s12938-020-00807-x

31 He K, Zhao W, Xie X, *et al.* Synergistic learning of lung lobe segmentation and hierarchical multi-instance classification for automated severity assessment of COVID-19 in CT images. *Pattern Recognit* 2021;**113**:107828. doi:10.1016/j.patcog.2021.107828

32 Li Z, Zhao W, Shi F, *et al.* A novel multiple instance learning framework for COVID-19 severity assessment via data augmentation and self-supervised learning. *Med Image Anal* 2021;**69**:101978. doi:10.1016/j.media.2021.101978

33 Ning W, Lei S, Yang J, *et al.* Open resource of clinical data from patients with pneumonia for the prediction of COVID-19 outcomes via deep learning. *Nat Biomed Eng* 2020;**4**:1197–207. doi:10.1038/s41551-020-00633-5

34 Fang C, Bai S, Chen Q, *et al.* Deep learning for predicting COVID-19 malignant progression. *Medical image analysis* 2021;**72**. doi:10.1016/j.media.2021.102096

35 Shi W, Peng X, Liu T, *et al.* A deep learning-based quantitative computed tomography model for predicting the severity of COVID-19: a retrospective study of 196 patients. *Ann Transl Med* 2021;**9**:216. doi:10.21037/atm-20-2464

36 Chen W, Yao M, Zhu Z, *et al.* The application research of AI image recognition and processing technology in the early diagnosis of the COVID-19. *BMC medical imaging* 2022;**22**. doi:10.1186/s12880-022-00753-1

37 Xiao L-S, Li P, Sun F, *et al.* Development and Validation of a Deep Learning-Based Model Using Computed Tomography Imaging for Predicting Disease Severity of Coronavirus Disease 2019. *Front Bioeng Biotechnol* 2020;**8**:898. doi:10.3389/fbioe.2020.00898

38 Feng Z, Shen H, Gao K, *et al.* Machine learning based on clinical characteristics and chest CT quantitative measurements for prediction of adverse clinical outcomes in hospitalized patients with COVID-19. *European radiology* 2021;**31**:7925–35. doi:10.1007/s00330-021-07957-z

39 Carvalho ARS, Guimarães A, Werberich GM, *et al.* COVID-19 Chest Computed Tomography to Stratify Severity and Disease Extension by Artificial Neural Network Computer-Aided Diagnosis. *Front Med (Lausanne)* 2020;**7**:577609. doi:10.3389/fmed.2020.577609

40 Aboutalebi H, Pavlova M, Shafiee MJ, *et al.* COVID-Net CXR-S: Deep Convolutional Neural Network for Severity Assessment of COVID-19 Cases from Chest X-ray Images. *Diagnostics (Basel)* 2021;**12**:25. doi:10.3390/diagnostics12010025

41 Ho TT, Park J, Kim T, *et al.* Deep learning models for predicting severe progression in COVID-19-infected patients: Retrospective study. *JMIR medical informatics* 2021;**9**. doi:10.2196/24973

42 Udriștoiu AL, Ghenea AE, Udriștoiu Ștefan, *et al.* COVID-19 and Artificial Intelligence: An Approach to Forecast the Severity of Diagnosis. *Life (Basel)* 2021;**11**:1281. doi:10.3390/life11111281

43 Ortiz S, Rojas F, Valenzuela O, *et al.* Determination of the Severity and Percentage of COVID-19 Infection through a Hierarchical Deep Learning System. *J Pers Med* 2022;**12**:535. doi:10.3390/jpm12040535

44 Zhang K, Liu X, Shen J, *et al.* Clinically Applicable AI System for Accurate Diagnosis, Quantitative Measurements, and Prognosis of COVID-19 Pneumonia Using Computed Tomography. *Cell* 2020;**181**:1423-1433.e11. doi:10.1016/j.cell.2020.04.045

45 Jiao Z, Choi JW, Halsey K, *et al.* Prognostication of patients with COVID-19 using artificial intelligence based on chest x-rays and clinical data: a retrospective study. *Lancet Digit Health* 2021;**3**:e286–94. doi:10.1016/S2589-7500(21)00039-X

46 Kwon YJF, Toussie D, Finkelstein M, *et al.* Combining Initial Radiographs and Clinical Variables Improves Deep Learning Prognostication in Patients with COVID-19 from the Emergency Department. *Radiology Artificial intelligence* 2021;**3**:e200098. doi:10.1148/ryai.2020200098

47 Lassau N, Ammari S, Chouzenoux E, *et al.* Integrating deep learning CT-scan model, biological and clinical variables to predict severity of COVID-19 patients. *Nature Communications* 2021;**12**. doi:10.1038/s41467-020-20657-4

48 Soda P, D’Amico NC, Tessadori J, *et al.* AIforCOVID: Predicting the clinical outcomes in patients with COVID-19 applying AI to chest-X-rays. An Italian multicentre study. *Med Image Anal* 2021;**74**:102216. doi:10.1016/j.media.2021.102216

49 Chieregato M, Frangiamore F, Morassi M, *et al.* A hybrid machine learning/deep learning COVID-19 severity predictive model from CT images and clinical data. *Sci Rep* 2022;**12**:4329. doi:10.1038/s41598-022-07890-1

50 Wang R, Jiao Z, Yang L, *et al.* Artificial intelligence for prediction of COVID-19 progression using CT imaging and clinical data. *Eur Radiol* 2022;**32**:205–12. doi:10.1007/s00330-021-08049-8

68 Huang Y, Lu Y, Huang Y-M, *et al.* Obesity in patients with COVID-19: a systematic review and meta-analysis. *Metabolism* 2020;**113**:154378. doi:10.1016/j.metabol.2020.154378

69 Yang Y, Luo K, Jiang Y, *et al.* The Impact of Frailty on COVID-19 Outcomes: A Systematic Review and Meta-analysis of 16 Cohort Studies. *J Nutr Health Aging* 2021;**25**:702–9. doi:10.1007/s12603-021-1611-9

70 Fabião J, Sassi B, Pedrollo EF, *et al.* Why do men have worse COVID-19-related outcomes? A systematic review and meta-analysis with sex adjusted for age. *Braz J Med Biol Res* 2022;**55**:e11711. doi:10.1590/1414-431X2021e11711

71 Lazarus G, Audrey J, Wangsaputra VK, *et al.* High admission blood glucose independently predicts poor prognosis in COVID-19 patients: A systematic review and dose-response meta-analysis. *Diabetes Res Clin Pract* 2021;**171**:108561. doi:10.1016/j.diabres.2020.108561

72 Du Y, Zhou N, Zha W, *et al.* Hypertension is a clinically important risk factor for critical illness and mortality in COVID-19: A meta-analysis. *Nutr Metab Cardiovasc Dis* 2021;**31**:745–55. doi:10.1016/j.numecd.2020.12.009

73 Su W, Wang H, Sun C, *et al.* The Association Between Previous Influenza Vaccination and COVID-19 Infection Risk and Severity: A Systematic Review and Meta-analysis. *Am J Prev Med* 2022;**63**:121–30. doi:10.1016/j.amepre.2022.02.008

74 Váncsa S, Dembrovszky F, Farkas N, *et al.* Repeated SARS-CoV-2 Positivity: Analysis of 123 Cases. *Viruses* 2021;**13**:512. doi:10.3390/v13030512

75 Guan Y, Chen C, Guo A, *et al.* Prolonged symptom onset to admission time is associated with severe Coronavirus disease: A meta combined propensity-adjusted analysis. *J Med Virol* 2021;**93**:6714–21. doi:10.1002/jmv.27253

76 Feikin DR, Higdon MM, Abu-Raddad LJ, *et al.* Duration of effectiveness of vaccines against SARS-CoV-2 infection and COVID-19 disease: results of a systematic review and meta-regression. *Lancet* 2022;**399**:924–44. doi:10.1016/S0140-6736(22)00152-0

77 Földi M, Farkas N, Kiss S, *et al.* Visceral Adiposity Elevates the Risk of Critical Condition in COVID-19: A Systematic Review and Meta-Analysis. *Obesity (Silver Spring)* 2021;**29**:521–8. doi:10.1002/oby.23096

78 Romero Starke K, Reissig D, Petereit-Haack G, *et al.* The isolated effect of age on the risk of COVID-19 severe outcomes: a systematic review with meta-analysis. *BMJ Glob Health* 2021;**6**:e006434. doi:10.1136/bmjgh-2021-006434

79 Agyemang C, Richters A, Jolani S, *et al.* Ethnic minority status as social determinant for COVID-19 infection, hospitalisation, severity, ICU admission and deaths in the early phase of the pandemic: a meta-analysis. *BMJ Glob Health* 2021;**6**:e007433. doi:10.1136/bmjgh-2021-007433

80 Farsalinos K, Bagos PG, Giannouchos T, *et al.* Smoking prevalence among hospitalized COVID-19 patients and its association with disease severity and mortality: an expanded re-analysis of a recent publication. *Harm Reduct J* 2021;**18**:9. doi:10.1186/s12954-020-00437-5

81 Hou H, Li Y, Zhang P, *et al.* Smoking Is Independently Associated With an Increased Risk for COVID-19 Mortality: A Systematic Review and Meta-analysis Based on Adjusted Effect Estimates. *Nicotine Tob Res* 2021;**23**:1947–51. doi:10.1093/ntr/ntab112

82 Gatti M, Rinaldi M, Bussini L, *et al.* Clinical outcome in solid organ transplant recipients affected by COVID-19 compared to general population: a systematic review and meta-analysis. *Clin Microbiol Infect* 2022;**28**:1057–65. doi:10.1016/j.cmi.2022.02.039

83 Udomkarnjananun S, Kerr SJ, Townamchai N, *et al.* Mortality risk factors of COVID-19 infection in kidney transplantation recipients: a systematic review and meta-analysis of cohorts and clinical registries. *Sci Rep* 2021;**11**:20073. doi:10.1038/s41598-021-99713-y

84 Ulloque-Badaracco JR, Hernandez-Bustamante EA, Herrera-Añazco P, *et al.* Prognostic value of apolipoproteins in COVID-19 patients: A systematic review and meta-analysis. *Travel Med Infect Dis* 2021;**44**:102200. doi:10.1016/j.tmaid.2021.102200

85 Zinellu A, Sotgia S, Carru C, *et al.* B-Type Natriuretic Peptide Concentrations, COVID-19 Severity, and Mortality: A Systematic Review and Meta-Analysis With Meta-Regression. *Front Cardiovasc Med* 2021;**8**:690790. doi:10.3389/fcvm.2021.690790

86 Li X, Pan X, Li Y, *et al.* Cardiac injury associated with severe disease or ICU admission and death in hospitalized patients with COVID-19: a meta-analysis and systematic review. *Crit Care* 2020;**24**:468. doi:10.1186/s13054-020-03183-z

87 Wungu CDK, Khaerunnisa S, Putri EAC, *et al.* Meta-analysis of cardiac markers for predictive factors on severity and mortality of COVID-19. *Int J Infect Dis* 2021;**105**:551–9. doi:10.1016/j.ijid.2021.03.008

88 Martha JW, Wibowo A, Pranata R. Prognostic value of elevated lactate dehydrogenase in patients with COVID-19: a systematic review and meta-analysis. *Postgrad Med J* 2022;**98**:422–7. doi:10.1136/postgradmedj-2020-139542

89 Toraih EA, Elshazli RM, Hussein MH, *et al.* Association of cardiac biomarkers and comorbidities with increased mortality, severity, and cardiac injury in COVID-19 patients: A meta-regression and decision tree analysis. *J Med Virol* 2020;**92**:2473–88. doi:10.1002/jmv.26166

90 Zhao R, Su Z, Komissarov AA, *et al.* Associations of D-Dimer on Admission and Clinical Features of COVID-19 Patients: A Systematic Review, Meta-Analysis, and Meta-Regression. *Front Immunol* 2021;**12**:691249. doi:10.3389/fimmu.2021.691249

91 Lin J, Yan H, Chen H, *et al.* COVID-19 and coagulation dysfunction in adults: A systematic review and meta-analysis. *J Med Virol* 2021;**93**:934–44. doi:10.1002/jmv.26346

92 Zinellu A, Paliogiannis P, Carru C, *et al.* INR and COVID-19 severity and mortality: A systematic review with meta-analysis and meta-regression. *Adv Med Sci* 2021;**66**:372–80. doi:10.1016/j.advms.2021.07.009

93 Lippi G, Henry BM, Favaloro EJ. Mean Platelet Volume Predicts Severe COVID-19 Illness. *Semin Thromb Hemost* 2021;**47**:456–9. doi:10.1055/s-0041-1727283

94 Mitra S, Ling RR, Yang IX, *et al.* Severe COVID-19 and coagulopathy: A systematic review and meta-analysis. *Ann Acad Med Singap* 2021;**50**:325–35.

95 Chang Y, Bai M, You Q. Associations between Serum Interleukins (IL-1β, IL-2, IL-4, IL-6, IL-8, and IL-10) and Disease Severity of COVID-19: A Systematic Review and Meta-Analysis. *Biomed Res Int* 2022;**2022**:2755246. doi:10.1155/2022/2755246

96 Udomsinprasert W, Jittikoon J, Sangroongruangsri S, *et al.* Circulating Levels of Interleukin-6 and Interleukin-10, But Not Tumor Necrosis Factor-Alpha, as Potential Biomarkers of Severity and Mortality for COVID-19: Systematic Review with Meta-analysis. *J Clin Immunol* 2021;**41**:11–22. doi:10.1007/s10875-020-00899-z

97 Wibowo A, Pranata R, Lim MA, *et al.* Endotheliopathy marked by high von Willebrand factor (vWF) antigen in COVID-19 is associated with poor outcome: a systematic review and meta-analysis. *Int J Infect Dis* 2022;**117**:267–73. doi:10.1016/j.ijid.2021.06.051

98 Xu X, Feng Y, Jia Y, *et al.* Prognostic value of von Willebrand factor and ADAMTS13 in patients with COVID-19: A systematic review and meta-analysis. *Thromb Res* 2022;**218**:83–98. doi:10.1016/j.thromres.2022.08.017

99 Yamada T, Wakabayashi M, Yamaji T, *et al.* Value of leukocytosis and elevated C-reactive protein in predicting severe coronavirus 2019 (COVID-19): A systematic review and meta-analysis. *Clin Chim Acta* 2020;**509**:235–43. doi:10.1016/j.cca.2020.06.008

100 Huang R, Xie L, He J, *et al.* Association between the peripheral blood eosinophil counts and COVID-19: A meta-analysis. *Medicine (Baltimore)* 2021;**100**:e26047. doi:10.1097/MD.0000000000026047

101 Huang G, Kovalic AJ, Graber CJ. Prognostic Value of Leukocytosis and Lymphopenia for Coronavirus Disease Severity. *Emerg Infect Dis* 2020;**26**:1839–41. doi:10.3201/eid2608.201160

102 Sarkar S, Khanna P, Singh AK. The Impact of Neutrophil-Lymphocyte Count Ratio in COVID-19: A Systematic Review and Meta-Analysis. *J Intensive Care Med* 2022;**37**:857–69. doi:10.1177/08850666211045626

103 Zhou S, Li H, Li S. The Associations of Iron Related Biomarkers with Risk, Clinical Severity and Mortality in SARS-CoV-2 Patients: A Meta-Analysis. *Nutrients* 2022;**14**:3406. doi:10.3390/nu14163406

104 Kovalic AJ, Huang G, Thuluvath PJ, *et al.* Elevated Liver Biochemistries in Hospitalized Chinese Patients With Severe COVID-19: Systematic Review and Meta-analysis. *Hepatology* 2021;**73**:1521–30. doi:10.1002/hep.31472

105 Mattiuzzi C, Lippi G. Serum prealbumin values predict the severity of coronavirus disease 2019 (COVID-19). *J Med Virol* 2021;**93**:620–1. doi:10.1002/jmv.26385

106 Zinellu A, Mangoni AA. Serum Complement C3 and C4 and COVID-19 Severity and Mortality: A Systematic Review and Meta-Analysis With Meta-Regression. *Front Immunol* 2021;**12**:696085. doi:10.3389/fimmu.2021.696085

107 Zinellu A, Mangoni AA. Cystatin C, COVID-19 severity and mortality: a systematic review and meta-analysis. *J Nephrol* 2022;**35**:59–68. doi:10.1007/s40620-021-01139-2

108 Llamas M, Garo ML, Giovanella L. Low free-T3 serum levels and prognosis of COVID-19: systematic review and meta-analysis. *Clin Chem Lab Med* 2021;**59**:1906–13. doi:10.1515/cclm-2021-0805

109 Li Y, Wei L, He L, *et al.* Interferon-induced transmembrane protein 3 gene polymorphisms are associated with COVID-19 susceptibility and severity: A meta-analysis. *J Infect* 2022;**84**:825–33. doi:10.1016/j.jinf.2022.04.029

110 Song G, Qiao W, Wang X, *et al.* Association of Lung Ultrasound Score with Mortality and Severity of COVID-19: A Meta-Analysis and Trial Sequential Analysis. *Int J Infect Dis* 2021;**108**:603–9. doi:10.1016/j.ijid.2021.06.026

111 Sarkar S, Kannan S, Khanna P, *et al.* Role of red blood cell distribution width, as a prognostic indicator in COVID-19: A systematic review and meta-analysis. *Rev Med Virol* 2022;**32**:e2264. doi:10.1002/rmv.2264

112 Zinellu A, Paliogiannis P, Carru C, *et al.* Serum amyloid A concentrations, COVID-19 severity and mortality: An updated systematic review and meta-analysis. *Int J Infect Dis* 2021;**105**:668–74. doi:10.1016/j.ijid.2021.03.025

113 Petrović T, Vijay A, Vučković F, *et al.* IgG N-glycome changes during the course of severe COVID-19: An observational study. *EBioMedicine* 2022;**81**:104101. doi:10.1016/j.ebiom.2022.104101

114 Zhang A, Leng Y, Zhang Y, *et al.* Meta-analysis of coagulation parameters associated with disease severity and poor prognosis of COVID-19. *Int J Infect Dis* 2020;**100**:441–8. doi:10.1016/j.ijid.2020.09.021

115 Rangel-Ramírez VV, Macías-Piña KA, Servin-Garrido RR, *et al.* A systematic review and meta-analysis of the IgA seroprevalence in COVID-19 patients: Is there a role for IgA in COVID-19 diagnosis or severity? *Microbiol Res* 2022;**263**:127105. doi:10.1016/j.micres.2022.127105

116 Lagunas-Rangel FA, Chávez-Valencia V. High IL-6/IFN-γ ratio could be associated with severe disease in COVID-19 patients. *J Med Virol* 2020;**92**:1789–90. doi:10.1002/jmv.25900

117 Vazzana N, Dipaola F, Ognibene S. Procalcitonin and secondary bacterial infections in COVID-19: association with disease severity and outcomes. *Acta Clin Belg* 2022;**77**:268–72. doi:10.1080/17843286.2020.1824749

118 Mutneja HR, Bhurwal A, Arora S, *et al.* Acute pancreatitis in patients with COVID-19 is more severe and lethal: a systematic review and meta-analysis. *Scand J Gastroenterol* 2021;**56**:1467–72. doi:10.1080/00365521.2021.1971757

119 Wang B, Luo Q, Zhang W, *et al.* The Involvement of Chronic Kidney Disease and Acute Kidney Injury in Disease Severity and Mortality in Patients with COVID-19: A Meta-Analysis. *Kidney Blood Press Res* 2021;**46**:17–30. doi:10.1159/000512211

120 Shao M, Li X, Liu F, *et al.* Acute kidney injury is associated with severe infection and fatality in patients with COVID-19: A systematic review and meta-analysis of 40 studies and 24,527 patients. *Pharmacol Res* 2020;**161**:105107. doi:10.1016/j.phrs.2020.105107

121 Ouyang L, Gong Y, Zhu Y, *et al.* Association of acute kidney injury with the severity and mortality of SARS-CoV-2 infection: A meta-analysis. *Am J Emerg Med* 2021;**43**:149–57. doi:10.1016/j.ajem.2020.08.089

122 Harapan H, Fajar JK, Supriono S, *et al.* The prevalence, predictors and outcomes of acute liver injury among patients with COVID-19: A systematic review and meta-analysis. *Rev Med Virol* 2022;**32**:e2304. doi:10.1002/rmv.2304

123 Khoury E, Nevitt S, Madsen WR, *et al.* Differences in Outcomes and Factors Associated With Mortality Among Patients With SARS-CoV-2 Infection and Cancer Compared With Those Without Cancer: A Systematic Review and Meta-analysis. *JAMA Netw Open* 2022;**5**:e2210880. doi:10.1001/jamanetworkopen.2022.10880

124 Yu J-N, Wu B-B, Yang J, *et al.* Cardio-Cerebrovascular Disease is Associated With Severity and Mortality of COVID-19: A Systematic Review and Meta-Analysis. *Biol Res Nurs* 2021;**23**:258–69. doi:10.1177/1099800420951984

125 Gülsen A, König IR, Jappe U, *et al.* Effect of comorbid pulmonary disease on the severity of COVID-19: A systematic review and meta-analysis. *Respirology* 2021;**26**:552–65. doi:10.1111/resp.14049

126 G A, I C, S A, *et al.* Association of Cardiovascular Disease With Coronavirus Disease 2019 (COVID-19) Severity: A Meta-Analysis. *Current problems in cardiology* 2020;**45**. doi:10.1016/j.cpcardiol.2020.100617

127 Matsushita K, Ding N, Kou M, *et al.* The Relationship of COVID-19 Severity with Cardiovascular Disease and Its Traditional Risk Factors: A Systematic Review and Meta-Analysis. *Glob Heart* 2020;**15**:64. doi:10.5334/gh.814

128 Mantovani A, Byrne CD, Zheng M-H, *et al.* Diabetes as a risk factor for greater COVID-19 severity and in-hospital death: A meta-analysis of observational studies. *Nutr Metab Cardiovasc Dis* 2020;**30**:1236–48. doi:10.1016/j.numecd.2020.05.014

129 Corona G, Pizzocaro A, Vena W, *et al.* Diabetes is most important cause for mortality in COVID-19 hospitalized patients: Systematic review and meta-analysis. *Rev Endocr Metab Disord* 2021;**22**:275–96. doi:10.1007/s11154-021-09630-8

130 Schlesinger S, Neuenschwander M, Lang A, *et al.* Risk phenotypes of diabetes and association with COVID-19 severity and death: a living systematic review and meta-analysis. *Diabetologia* 2021;**64**:1480–91. doi:10.1007/s00125-021-05458-8

131 Kaminska H, Szarpak L, Kosior D, *et al.* Impact of diabetes mellitus on in-hospital mortality in adult patients with COVID-19: a systematic review and meta-analysis. *Acta Diabetol* 2021;**58**:1101–10. doi:10.1007/s00592-021-01701-1

132 Yang W, Sun X, Zhang J, *et al.* The effect of metformin on mortality and severity in COVID-19 patients with diabetes mellitus. *Diabetes Res Clin Pract* 2021;**178**:108977. doi:10.1016/j.diabres.2021.108977

133 Jin S, Jin Y, Xu B, *et al.* Prevalence and Impact of Coagulation Dysfunction in COVID-19 in China: A Meta-Analysis. *Thromb Haemost* 2020;**120**:1524–35. doi:10.1055/s-0040-1714369

134 Zhou X, Cheng Z, Luo L, *et al.* Incidence and impact of disseminated intravascular coagulation in COVID-19 a systematic review and meta-analysis. *Thromb Res* 2021;**201**:23–9. doi:10.1016/j.thromres.2021.02.010

135 Liu Y, Pan Y, Yin Y, *et al.* Association of dyslipidemia with the severity and mortality of coronavirus disease 2019 (COVID-19): a meta-analysis. *Virol J* 2021;**18**:157. doi:10.1186/s12985-021-01604-1

136 Siow I, Lee KS, Zhang JJY, *et al.* Encephalitis as a neurological complication of COVID-19: A systematic review and meta-analysis of incidence, outcomes, and predictors. *Eur J Neurol* 2021;**28**:3491–502. doi:10.1111/ene.14913

137 Naimi A, Yashmi I, Jebeleh R, *et al.* Comorbidities and mortality rate in COVID-19 patients with hematological malignancies: A systematic review and meta-analysis. *J Clin Lab Anal* 2022;**36**:e24387. doi:10.1002/jcla.24387

138 Yonas E, Alwi I, Pranata R, *et al.* Effect of heart failure on the outcome of COVID-19 - A meta analysis and systematic review. *Am J Emerg Med* 2021;**46**:204–11. doi:10.1016/j.ajem.2020.07.009

139 Song HJJMD, Chia AZQ, Tan BKJ, *et al.* Electrolyte imbalances as poor prognostic markers in COVID-19: a systemic review and meta-analysis. *J Endocrinol Invest* Published Online First: 7 September 2022. doi:10.1007/s40618-022-01877-5

140 Pan L, Huang P, Xie X, *et al.* Metabolic associated fatty liver disease increases the severity of COVID-19: A meta-analysis. *Dig Liver Dis* 2021;**53**:153–7. doi:10.1016/j.dld.2020.09.007

141 Hariyanto TI, Kurniawan A. Obstructive sleep apnea (OSA) and outcomes from coronavirus disease 2019 (COVID-19) pneumonia: a systematic review and meta-analysis. *Sleep Med* 2021;**82**:47–53. doi:10.1016/j.sleep.2021.03.029

142 Rathore SS, Hussain N, Manju AH, *et al.* Prevalence and clinical outcomes of pleural effusion in COVID-19 patients: A systematic review and meta-analysis. *J Med Virol* 2022;**94**:229–39. doi:10.1002/jmv.27301

143 Corica B, Marra AM, Basili S, *et al.* Prevalence of right ventricular dysfunction and impact on all-cause death in hospitalized patients with COVID-19: a systematic review and meta-analysis. *Sci Rep* 2021;**11**:17774. doi:10.1038/s41598-021-96955-8

144 Tang K, Wu L, Luo Y, *et al.* Quantitative assessment of SARS-CoV-2 RNAemia and outcome in patients with coronavirus disease 2019. *J Med Virol* 2021;**93**:3165–75. doi:10.1002/jmv.26876

145 Li S, Ren J, Hou H, *et al.* The association between stroke and COVID-19-related mortality: a systematic review and meta-analysis based on adjusted effect estimates. *Neurol Sci* 2022;**43**:4049–59. doi:10.1007/s10072-022-06024-9

146 Srivastava R, Parveen R, Mishra P, *et al.* Venous thromboembolism is linked to severity of disease in COVID-19 patients: A systematic literature review and exploratory meta-analysis. *Int J Clin Pract* 2021;**75**:e14910. doi:10.1111/ijcp.14910

147 Gao Y, Chen Y, Liu M, *et al.* Nervous system diseases are associated with the severity and mortality of patients with COVID-19: a systematic review and meta-analysis. *Epidemiol Infect* 2021;**149**:e66. doi:10.1017/S0950268821000376

148 Hariyanto TI, Putri C, Hananto JE, *et al.* Delirium is a good predictor for poor outcomes from coronavirus disease 2019 (COVID-19) pneumonia: A systematic review, meta-analysis, and meta-regression. *J Psychiatr Res* 2021;**142**:361–8. doi:10.1016/j.jpsychires.2021.08.031

149 Hariyanto TI, Putri C, Arisa J, *et al.* Dementia and outcomes from coronavirus disease 2019 (COVID-19) pneumonia: A systematic review and meta-analysis. *Arch Gerontol Geriatr* 2021;**93**:104299. doi:10.1016/j.archger.2020.104299

150 Siahaan YMT, Ketaren RJ, Hartoyo V, *et al.* Epilepsy and the risk of severe coronavirus disease 2019 outcomes: A systematic review, meta-analysis, and meta-regression. *Epilepsy Behav* 2021;**125**:108437. doi:10.1016/j.yebeh.2021.108437

151 Vai B, Mazza MG, Delli Colli C, *et al.* Mental disorders and risk of COVID-19-related mortality, hospitalisation, and intensive care unit admission: a systematic review and meta-analysis. *Lancet Psychiatry* 2021;**8**:797–812. doi:10.1016/S2215-0366(21)00232-7

152 Sunjaya AP, Allida SM, Di Tanna GL, *et al.* Asthma and COVID-19 risk: a systematic review and meta-analysis. *Eur Respir J* 2022;**59**:2101209. doi:10.1183/13993003.01209-2021

153 Akiyama S, Hamdeh S, Micic D, *et al.* Prevalence and clinical outcomes of COVID-19 in patients with autoimmune diseases: a systematic review and meta-analysis. *Ann Rheum Dis* 2021;**80**:384–91. doi:10.1136/annrheumdis-2020-218946

154 Moonla C, Sosothikul D, Chiasakul T, *et al.* Anticoagulation and In-Hospital Mortality From Coronavirus Disease 2019: A Systematic Review and Meta-Analysis. *Clin Appl Thromb Hemost* 2021;**27**:10760296211009000. doi:10.1177/10760296211008999

155 Hariyanto TI, Lugito NPH, Yanto TA, *et al.* Insulin Therapy and Outcome of Coronavirus Disease 2019 (COVID-19): A Systematic Review, Meta-Analysis, and Meta-Regression. *Endocr Metab Immune Disord Drug Targets* 2022;**22**:481–9. doi:10.2174/1871530321666210709164925

156 Ao G, Li A, Wang Y, *et al.* Opioid usage and COVID-19 prognosis: A systematic review and meta-analysis. *Am J Emerg Med* 2022;**56**:51–6. doi:10.1016/j.ajem.2022.03.048

157 Diaz-Arocutipa C, Melgar-Talavera B, Alvarado-Yarasca Á, *et al.* Statins reduce mortality in patients with COVID-19: an updated meta-analysis of 147 824 patients. *Int J Infect Dis* 2021;**110**:374–81. doi:10.1016/j.ijid.2021.08.004

158 Fernando ME, Drovandi A, Golledge J. Meta-analysis of the association between angiotensin pathway inhibitors and COVID-19 severity and mortality. *Syst Rev* 2021;**10**:243. doi:10.1186/s13643-021-01802-6

159 Ren L, Yu S, Xu W, *et al.* Lack of association of antihypertensive drugs with the risk and severity of COVID-19: A meta-analysis. *J Cardiol* 2021;**77**:482–91. doi:10.1016/j.jjcc.2020.10.015

160 Yekedüz E, Utkan G, Ürün Y. A systematic review and meta-analysis: the effect of active cancer treatment on severity of COVID-19. *Eur J Cancer* 2020;**141**:92–104. doi:10.1016/j.ejca.2020.09.028

161 Lin Z, Chen J, Han S. Impact of anti-cancer therapy on disease severity and mortality in cancer patients with COVID-19: a systematic review and meta-analysis. *Expert Rev Anticancer Ther* 2021;**21**:1055–66. doi:10.1080/14737140.2021.1927721

162 Yang Z, Liu J, Zhou Y, *et al.* The effect of corticosteroid treatment on patients with coronavirus infection: a systematic review and meta-analysis. *J Infect* 2020;**81**:e13–20. doi:10.1016/j.jinf.2020.03.062

163 Ebrahimi Chaharom F, Pourafkari L, Ebrahimi Chaharom AA, *et al.* Effects of corticosteroids on Covid-19 patients: A systematic review and meta-analysis on clinical outcomes. *Pulm Pharmacol Ther* 2022;**72**:102107. doi:10.1016/j.pupt.2021.102107

164 Israelsen SB, Ernst MT, Lundh A, *et al.* Proton Pump Inhibitor Use Is Not Strongly Associated With SARS-CoV-2 Related Outcomes: A Nationwide Study and Meta-analysis. *Clin Gastroenterol Hepatol* 2021;**19**:1845-1854.e6. doi:10.1016/j.cgh.2021.05.011

165 Hariyanto TI, Kurniawan A. Statin and outcomes of coronavirus disease 2019 (COVID-19): A systematic review, meta-analysis, and meta-regression. *Nutr Metab Cardiovasc Dis* 2021;**31**:1662–70. doi:10.1016/j.numecd.2021.02.020

166 Tac S, N S, G A, *et al.* Tocilizumab in COVID-19: a meta-analysis, trial sequential analysis, and meta-regression of randomized-controlled trials. *Intensive care medicine* 2021;**47**. doi:10.1007/s00134-021-06416-z

167 Ao G, Li J, Yuan Y, *et al.* Intravenous vitamin C use and risk of severity and mortality in COVID-19: A systematic review and meta-analysis. *Nutr Clin Pract* 2022;**37**:274–81. doi:10.1002/ncp.10832

168 Bassatne A, Basbous M, Chakhtoura M, *et al.* The link between COVID-19 and VItamin D (VIVID): A systematic review and meta-analysis. *Metabolism* 2021;**119**:154753. doi:10.1016/j.metabol.2021.154753

169 Wang Z, Joshi A, Leopold K, *et al.* Association of vitamin D deficiency with COVID-19 infection severity: Systematic review and meta-analysis. *Clin Endocrinol (Oxf)* 2022;**96**:281–7. doi:10.1111/cen.14540
